# Airway proteolytic control of pneumococcal competence

**DOI:** 10.1371/journal.ppat.1011421

**Published:** 2023-05-31

**Authors:** Haley Echlin, Amy Iverson, Ugo Sardo, Jason W. Rosch

**Affiliations:** Department of Infectious Diseases, St Jude Children’s Research Hospital, Memphis, Tennessee, United States of America; The University of Alabama at Birmingham, UNITED STATES

## Abstract

*Streptococcus pneumoniae* is an opportunistic pathogen that colonizes the upper respiratory tract asymptomatically and, upon invasion, can lead to severe diseases including otitis media, sinusitis, meningitis, bacteremia, and pneumonia. One of the first lines of defense against pneumococcal invasive disease is inflammation, including the recruitment of neutrophils to the site of infection. The invasive pneumococcus can be cleared through the action of serine proteases generated by neutrophils. It is less clear how serine proteases impact non-invasive pneumococcal colonization, which is the key first step to invasion and transmission. One significant aspect of pneumococcal biology and adaptation in the respiratory tract is its natural competence, which is triggered by a small peptide CSP. In this study, we investigate if serine proteases are capable of degrading CSP and the impact this has on pneumococcal competence. We found that CSP has several potential sites for trypsin-like serine protease degradation and that there were preferential cleavage sites recognized by the proteases. Digestion of CSP with two different trypsin-like serine proteases dramatically reduced competence in a dose-dependent manner. Incubation of CSP with mouse lung homogenate also reduced recombination frequency of the pneumococcus. These *ex vivo* experiments suggested that serine proteases in the lower respiratory tract reduce pneumococcal competence. This was subsequently confirmed measuring *in vivo* recombination frequencies after induction of protease production via poly (I:C) stimulation and via co-infection with influenza A virus, which dramatically lowered recombination events. These data shed light on a new mechanism by which the host can modulate pneumococcal behavior and genetic exchange via direct degradation of the competence signaling peptide.

## Author summary

*Streptococcus pneumoniae* is a major human pathogen that usually colonizes the nasopharynx asymptomatically, but can progress to disease causing meningitis, bacteremia, otitis media, and sinusitis. In response to invasive pneumococci, the host produces an inflammatory response, including the influx of serine proteases. Invasive *S*. *pneumoniae* can be cleared through the action of serine proteases, however little is known on how serine proteases impact *S*. *pneumoniae* processes during colonization. One key modulator of pneumococcal survival in the host environment is its innate competence to uptake DNA. Here, we investigate how serine proteases impact pneumococcal competence through modulation of a key signaling peptide, CSP. We show that two different recombinant serine proteases degrade CSPs and this degradation has a direct impact on pneumococcal competence. We further demonstrate a reduction in pneumococcal competence upon CSP incubation with mouse lung homogenates, which suggests that serine proteases produced in mouse lungs similarly degrade CSP. These data reveal a novel mechanism by which the host can modulate pneumococcal behavior and genetic exchange, including that required for the spread of antibiotic resistance, a growing threat as the number of cases involving multi-drug resistant pneumococci have increased worldwide.

## Introduction

*Streptococcus pneumoniae* is a major human pathogen causing a multitude of diseases ranging from sinusitis and otitis media to pneumonia, bacteremia, and meningitis [1]. While it is a considerable source of morbidity and mortality primarily in young children and the elderly populations, *S*. *pneumoniae* usually colonizes the upper respiratory tract asymptomatically in up to 65% of children and <10% of adults [2,3]. The balance of interactions between the pneumococcus and the host can impact the likelihood of transition from commensal to pathogen and the severity of the resulting disease [4]. The hallmark of pneumococcal invasive disease is inflammation and often heralds the influx of neutrophils, which are among the first cells recruited to the site of infection [5,6]. Two prominent mechanisms of neutrophil clearance are oxidative burst and production of antimicrobial molecules including serine proteases [7,8]. Serine proteases hydrolyze peptide bonds via the nucleophilic serine in the enzyme active site. Serine proteases are further classified by their substrate specificity, e.g., trypsin-like serine proteases prefer Arg and Lys residues [9–11]. Because of its antiphagocytic capsule, *S*. *pneumoniae* is less likely to be killed by neutrophils via oxidative burst and is instead cleared by serine proteases (such as elastase, cathepsin G, and protease 3) found in the azurophilic granules of neutrophils [7]. Besides direct degradation of pathogen proteins, neutrophilic serine proteases also play a role in activation of antimicrobials, including LL-37 [12].

While the impact of serine proteases produced in response to invasive disease has been studied considerably, the impact of host serine proteases on pneumococcal carriage and progression to disease in the respiratory tract is not as well understood. One group of serine proteases in the respiratory tract are the human airway trypsin-like proteases (HAT) which are part of the type II transmembrane family of serine proteases (TTSPs), the largest group of membrane-anchored serine proteases [13]. Originally identified in the sputum of patients with chronic airway diseases, HAT has been more recently shown to be found specifically on ciliated epithelial cells, rather than the basal cells, of the epithelial layer in the respiratory tract, suggesting a role within the epithelial layer and on the airway surface [13–15]. This is of particular interest in the context of pneumococcal interactions as asymptomatic colonization of *S*. *pneumoniae* requires adherence to the non-inflamed epithelial cells of the respiratory tract [2,16]. Once adhered to the respiratory tract surface, the pneumococcus must often compete with host factors (including serine proteases), changing environmental conditions, and other colonizing bacterial species [17]. Gaining insight into how the complex environment of the upper and lower respiratory tract impacts the strategies deployed by the pneumococcus is critical for understanding pneumococcal biology at the host-pathogen interface.

A powerful mechanism that *S*. *pneumoniae* utilizes to adapt to its changing environment is natural competence, a physiological state in which it undergoes active uptake of exogenous DNA [18,19]. Pneumococcal natural transformation is triggered via a quorum-sensing cascade induced at low population density of exponentially growing cells [20]. The competence cascade is triggered by a small pheromone—competence-stimulating peptide (CSP), which is encoded by *comC* and then processed and secreted as a mature 17-residue peptide. CSP activates the two-component regulatory system ComD (membrane receptor) and ComE (response regulator), which directly activates early competence genes including the *comAB* and *comCDE* operons and *comX*. ComAB is the membrane transporter that exports CSP to trigger cascades in other cells. Activation of *comCDE* provides a positive feedback loop to amplify the competence signal, while activation of *comX* promotes activation of late competence genes including those for DNA uptake [21–25]. Besides genes directly involved in the competence cascade, more than 100 genes are regulated upon activation of competence, including the genes involved in fratricide, in biofilm formation, and in virulence [26–32]. The complexity of how the pneumococcus adapts to changes in nutrients and competing cells, while thriving in the host, is not completely understood. However, it is imperative for us to understand the dynamics of this adaptation. Healthy children carrying *S*. *pneumoniae* could be colonized simultaneous by up to six different pneumococcal strains, occurring in up to 65% of young children [33–37]. This provides a high chance for acquisition of new genes, including those of antibiotic resistance. This is of particular concern as there has been a worldwide increase in the number of cases involving multi-drug resistant pneumococci [38].

In our current study, we investigate if the signaling peptide, CSP, can be degraded by trypsin-like serine proteases and how this impacts the recombination frequency of *Streptococcus pneumoniae*. We found that CSP was degraded by two trypsin-like serine proteases, including HAT. This digestion of CSP had a direct, dose-dependent impact on pneumococcal competence, whereby increased concentrations of trypsin-like serine proteases decreased recombination frequency. Modification of the peptide sequence of CSP1 suggested arginine at position nine to be a key modulator for the impact on recombination frequency. We also recapitulated the sensitivity of CSP to digestion by trypsin-like serine proteases *in vivo*. Taken together, these data shed light on a novel mechanism by which the host environment can modulate pneumococcal adaptation to its niche in the respiratory tract.

## Results

### CSPs are cleaved by trypsin-like serine proteases

**Fig 1:**
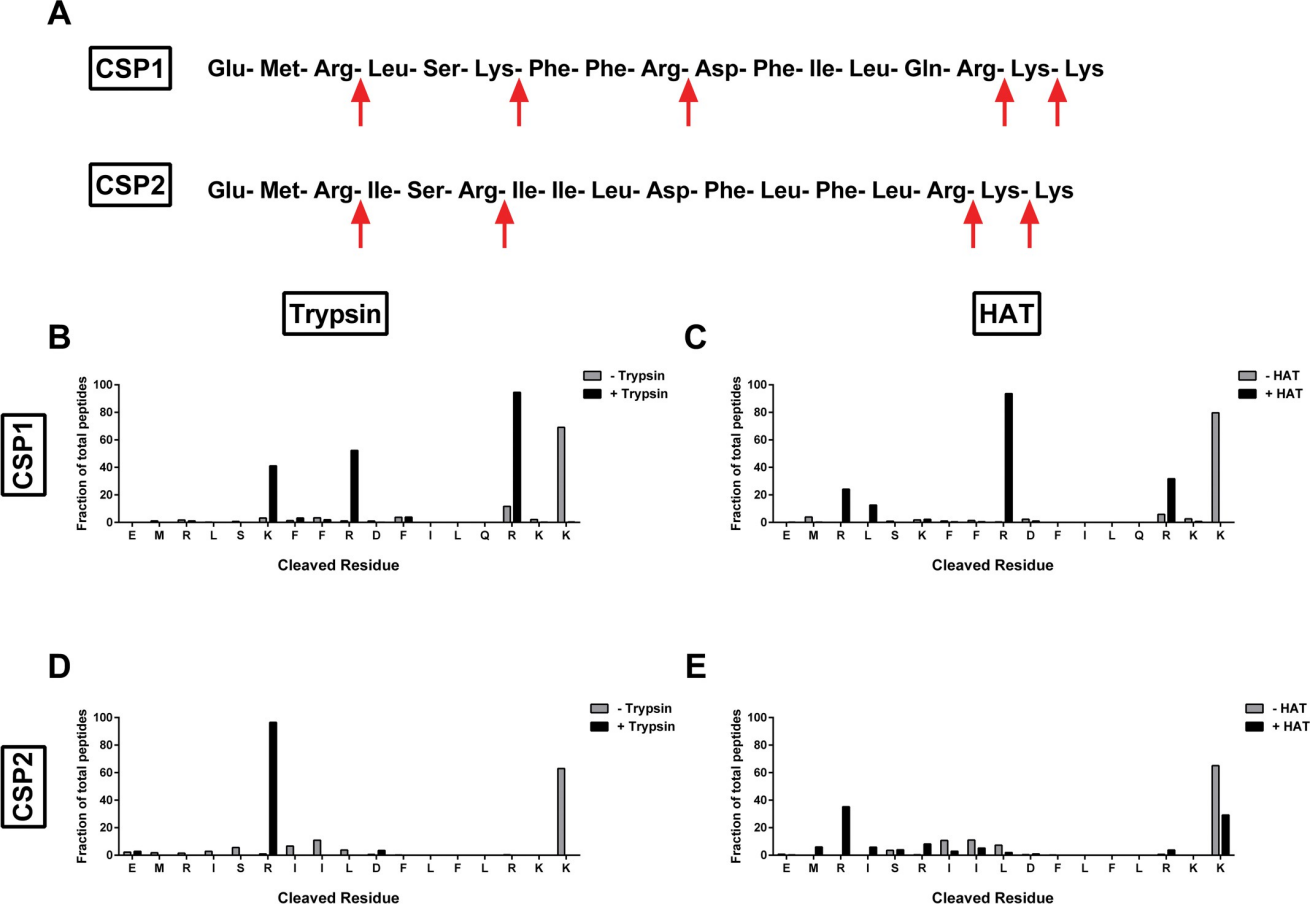
Serine proteases cleave competence-stimulating peptide of *S*. *pneumoniae*. **(A)** Sequence of competence-stimulating peptide 1 (CSP1) and peptide 2 (CSP2). Red arrows indicate potential cleavage sites by trypsin-like serine proteases. **(B)** Mass spectrometry analysis of cleaved residues of CSP1 upon incubation with no trypsin (grey) or with porcine trypsin (black) for 30 minutes. **(C)** Mass spectrometry analysis of cleaved residues of CSP1 upon incubation with no trypsin (grey) or with human airway trypsin (HAT) (black) for 30 minutes. **(D)** Mass spectrometry analysis of cleaved residues of CSP2 upon incubation with no trypsin (grey) or with porcine trypsin (black) for 30 minutes. **(E)** Mass spectrometry analysis of cleaved residues of CSP2 upon incubation with no trypsin (grey) or with HAT (black) for 30 minutes. **(B**-**E)** The fraction of each peptide detected by mass spectrometry was calculated by dividing the area of the cleaved peptide by the total area of all peptides. In the case of two cleavage sites on the same peptide, the fraction was equally attributed to both cleavage sites. Each bar represents the fraction of identified peptides that contained a cleavage event after the amino acid depicted under the bar. Bars above the final amino acid represent uncleaved CSP1 or CSP2.

Different strains of *S*. *pneumoniae* regulate their competence via competence stimulating peptides with strain-specific peptide sequences [39]. To investigate if CSPs could be cleaved by trypsin-like serine proteases, we analyzed the protein sequence of CSP1 and CSP2 [19] for potential cleavage sites. CSP1 contains five and CSP2 contains four potential trypsin cleavage sites (Fig 1A) after arginine or lysine [9]. To determine which sites are cleaved, CSP1 and CSP2 were incubated with either porcine trypsin or human airway trypsin-like protease (HAT; originally purified from patients with chronic airway disease). Mass spectrometry of the digested CSP1 demonstrated that CSP1 was cleaved by porcine trypsin at residues 6, 9, and 15 (Fig 1B) and by HAT at residues 3, 9, and 15 (Fig 1C). Interestingly, HAT digestion of CSP1 preferentially occurred at R9, while porcine trypsin digestion occurred primarily at R15 followed by K6 and R9. Like CSP1, CSP2 was cleaved by both porcine trypsin (Fig 1D) and HAT (Fig 1E). Unlike CSP1, porcine trypsin and HAT targeted a single site for cleavage—R6 for porcine trypsin and R3 for HAT. These data indicate that pneumococcal CSPs are both recognized and cleaved by serine proteases.

### Cleavage of CSPs by trypsin-like serine proteases reduces *S*. *pneumoniae* competence

**Fig 2:**
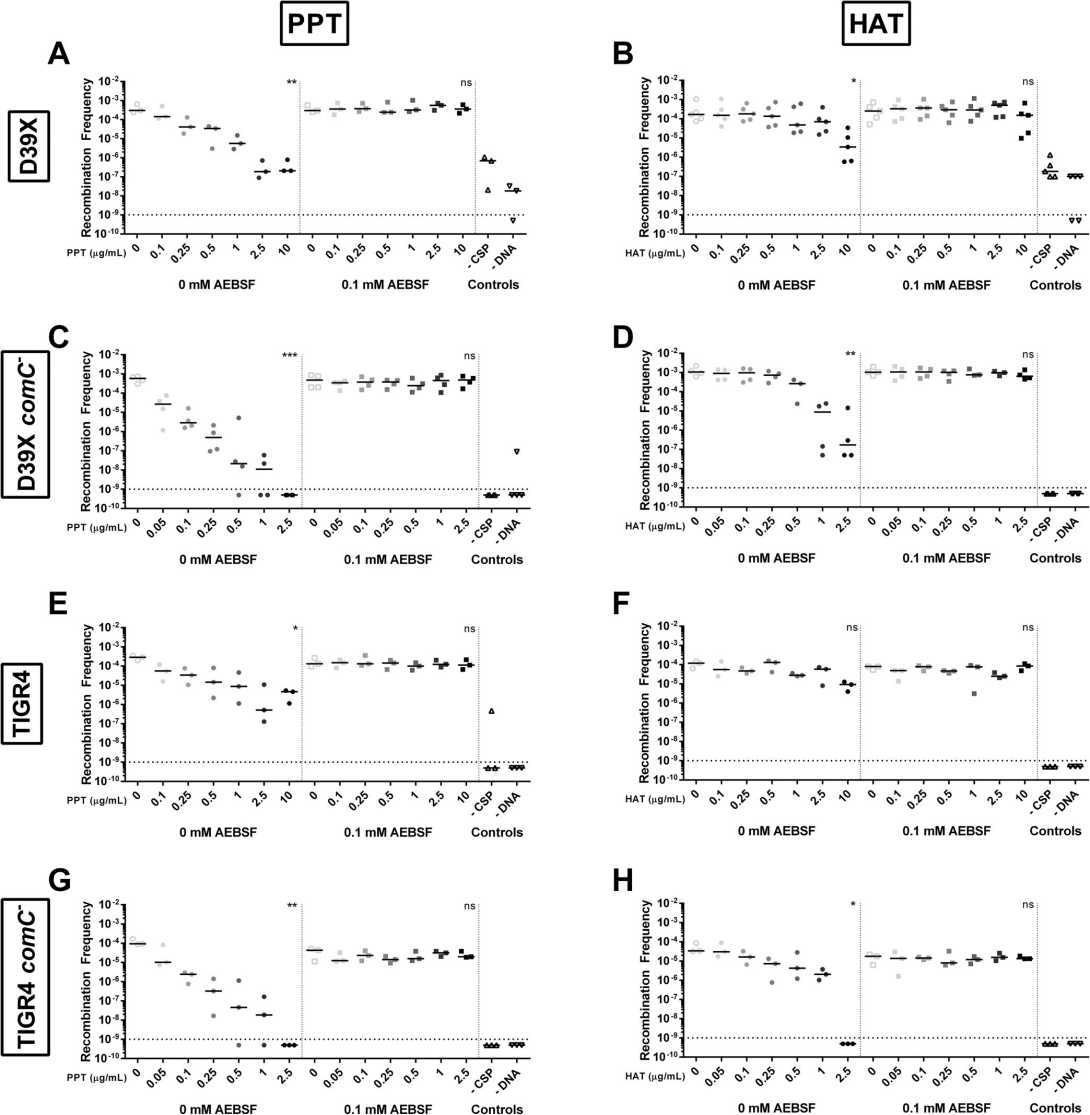
Serine proteases reduce recombination frequency of *S*. *pneumoniae* in a dose dependent manner. Recombination frequency upon incubation of CSP1 or CSP2 with increasing concentrations of serine proteases with and without 0.1mM of the serine protease inhibitor, AEBSF. D39x transformed with CSP1 incubated with **(A)** porcine pancreatic trypsin (PPT) or **(B)** human airway trypsin (HAT). D39x *comC*^-^ transformed with CSP1 incubated with **(C)** PPT or **(D)** HAT. TIGR4 transformed with CSP2 incubated with **(E)** PPT or **(F)** HAT. TIGR4 *comC*^-^ transformed with CSP2 incubated with **(G)** PPT or **(H)** HAT. Negative controls included no addition of CSP1 and no addition of gDNA. Lines represent median value. Dotted line represents lowest point of detection. Recombination frequencies of increasing concentrations of protease within each group (0 mM AEBSF or 0.1 mM AEBSF) were compared using Kruskal-Wallis one-way ANOVA; *p = 0.05–0.01, **p = 0.01–0.001, ***p = 0.001–0.0001.

As the CSPs were cleaved by trypsin-like serine proteases, we next ascertained if this cleavage impacts *S*. *pneumoniae* competence. CSP1 or CSP2 was incubated with increasing concentrations of trypsin-like proteases, porcine pancreatic trypsin (PPT) and HAT. The CSP1-trypsin mixture was used to transform D39x with genomic DNA from D39 Tn-seq library and successful recombinants were determined. The frequency of recombination was reduced by incubation of CSP1 with PPT (Fig 2A) and with HAT (Fig 2B) in a dose-dependent manner. To confirm that the observed reduced recombination frequency was due to trypsin cleavage of CSP1, the trypsin-like proteases were incubated with a serine protease inhibitor AEBSF prior to incubation with CSP1. Transformation with CSP1 incubated with inhibited PPT or HAT demonstrated similar recombination frequency compared to incubation with no trypsin, suggesting that the reduced competence was due to digestion of CSP1 by the serine proteases.

In this experimental setup, only exogenously added CSP1 would be exposed to trypsin-like proteases directly. Thus, any natively produced CSP1 could still induce competence. The presence of native CSP1 could explain why little difference was observed between 2.5 μg/mL and 10 ug/mL of PPT and why this recombination frequency was similar to the frequency of the control with no exogenous CSP1 added. To prevent potential induction of competence by native CSP1, a *comC* deletion strain that can no longer produce native CSP1 was generated, whereby transformation of this mutant relied exclusively on exogenously added peptide [25]. Recombination frequency of D39x *comC*^-^ was reduced when transformed with CSP1 incubated with PPT (Fig 2C) or HAT (Fig 2D). Similar to D39x, the frequency was reduced in a dose-dependent manner; however, the level of reduction was more dramatic in the *comC*^-^ mutant compared to the wild-type (Fig 2). Recombination frequency of D39x *comC*^-^ transformed with CSP1 incubated with PPT or HAT pre-incubated with inhibitor AEBSF was at a similar level as that of the no trypsin control.

To determine if cleavage of CSP2 impacts *S*. *pneumoniae* competence, CSP2 was incubated with increasing concentrations of trypsin-like proteases and this mixture was used to transform TIGR4 with genomic DNA from TIGR4 Tn-seq library. Like CSP1, the recombination frequency was reduced by incubation of CSP2 with PPT (Fig 2E) and, to a lesser extent, with HAT (Fig 2F) in a dose-dependent manner and this reduction was abrogated by incubating the trypsin-like proteases with inhibitor AEBSF. As observed in D39x, this loss in recombination frequency upon incubation with serine proteases was more pronounced in the TIGR4 *comC*^-^ strain that lacked the ability to produce native CSP2 (Fig 2G and 2H).

**Fig 3:**
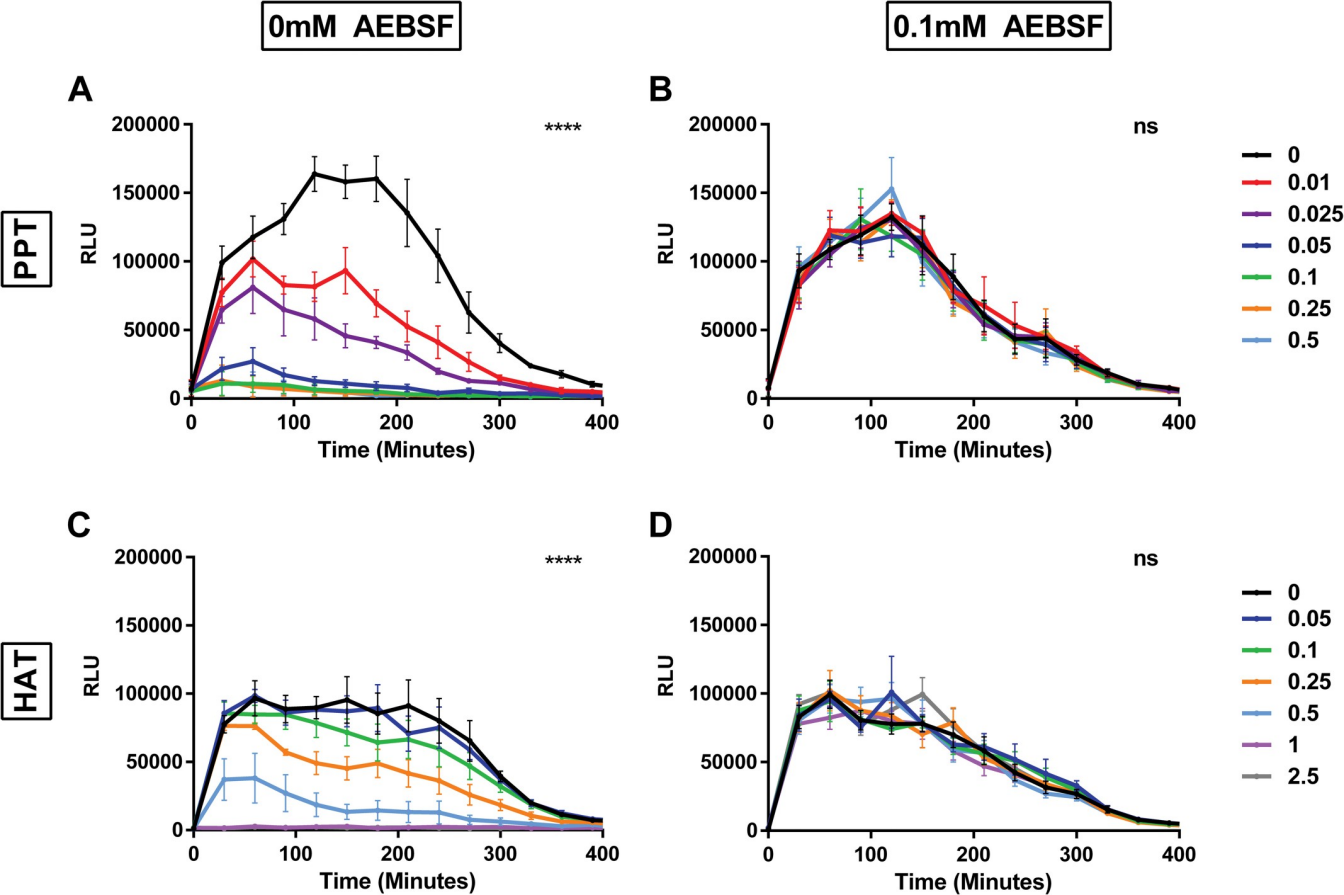
Serine proteases reduce expression of CSP induced luciferase in *S*. *pneumoniae*. Luminescence (RLU) of DLA3 grown in the presence of CSP1 incubated with increasing concentrations of serine proteases with and without inhibitor AEBSF. Incubation of CSP1 with PPT **(A)** without AEBSF or **(B)** with AEBSF. Incubation of CSP1 with HAT **(C)** without AEBSF or **(D)** with AEBSF. Experiment was repeated in triplicate. The mean value of RLU of each 30-minute timepoint is reported; error bars are SEM. Luminescence of increasing concentrations of protease within each group (0 mM AEBSF or 0.1 mM AEBSF) were compared using two-way ANOVA; ****p<0.0001.

To further confirm loss of competence upon CSP1 cleavage by trypsin-like proteases, CSP1 incubated with PPT or HAT was used to induce competence in DLA3, a strain derived from D39, and luminescence was measured (Fig 3). DLA3 contains a firefly luciferase reporter under the promoter of *ssbB* and activity of this reporter has been demonstrated to reflect competence and provides a way to monitor competence development in real time [40–42]. Luminescence of DLA3 was reduced in a dose-dependent manner upon incubation of CSP1 with PPT (Fig 3A) and this effect was abrogated if PPT was pre-incubated with AEBSF (Fig 3B). Incubation of CSP1 with HAT similarly reduced luminescence of DLA3 in a dose-dependent manner (Fig 3C and 3D). This loss of luminescence was not due to loss of growth (S1 Fig). Taken together, these results indicate that CSPs are cleaved by both PPT and HAT and this has a direct impact on the competence of *S*. *pneumoniae*.

### Preferential site of CSP cleavage is dependent on protease

**Fig 4:**
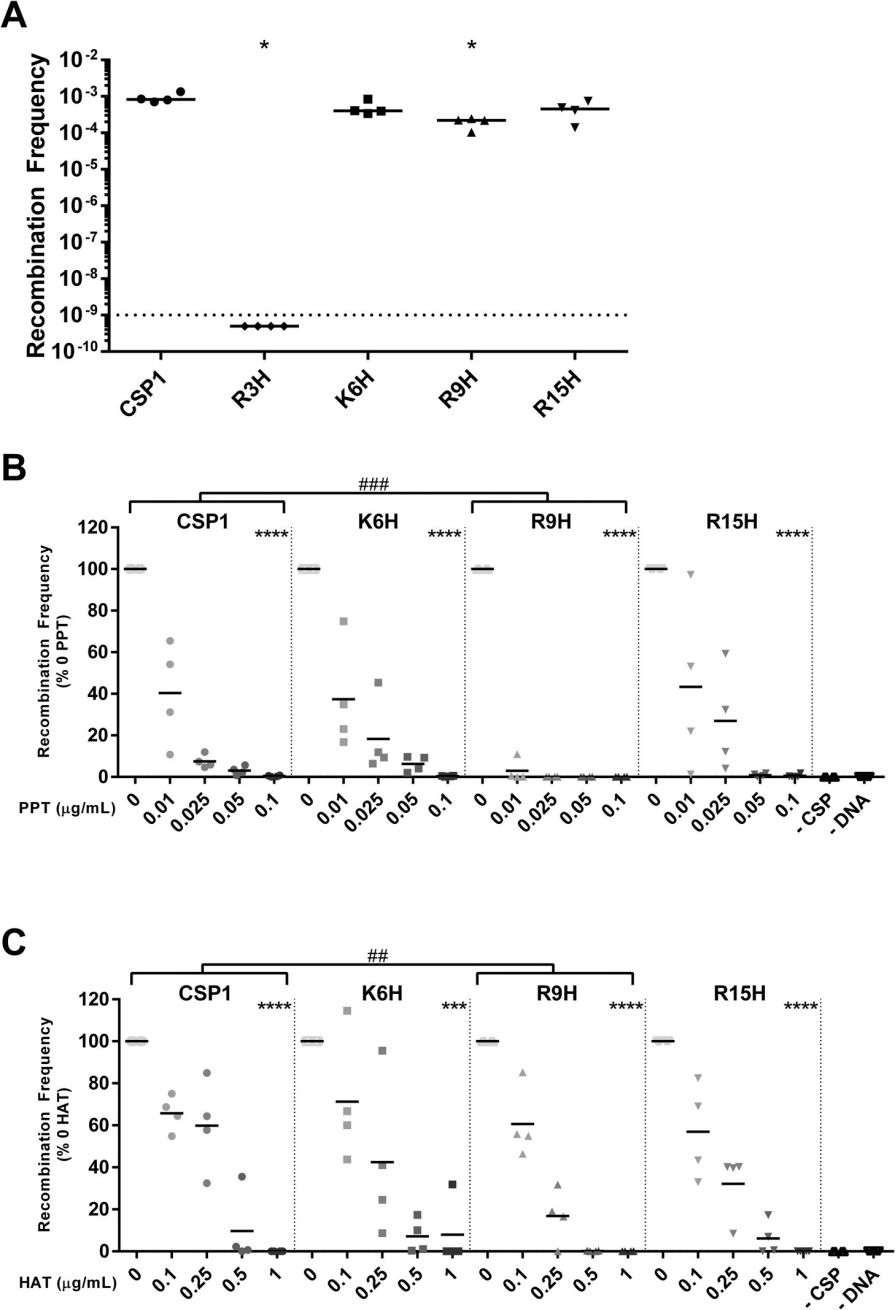
Modified CSP alters impact of protease on recombination frequency. Recombination frequency with modified CSP1. **(A)** Transformation of D39x *comC*^-^ with CSP1 with modifications R3H, K6H, R9H, and R15H. Transformation of D39x *comC*^-^ with modified CSP1 incubated with increasing concentrations of **(B)** PPT or **(C)** HAT. Recombination frequency reported as % of 0 trypsin. Negative controls included no addition of CSP1 and no addition of gDNA. Lines represent mean value. Recombination frequencies of increasing concentrations of protease within each modified CSP were compared using one-way ANOVA; ***p = 0.001–0.0001, ****p<0.0001. Changes in the concentrations of protease between modified CSP was compared using two-way ANOVA; ##p = 0.01–0.001, ###p = 0.001–0.0001.

**Fig 5:**
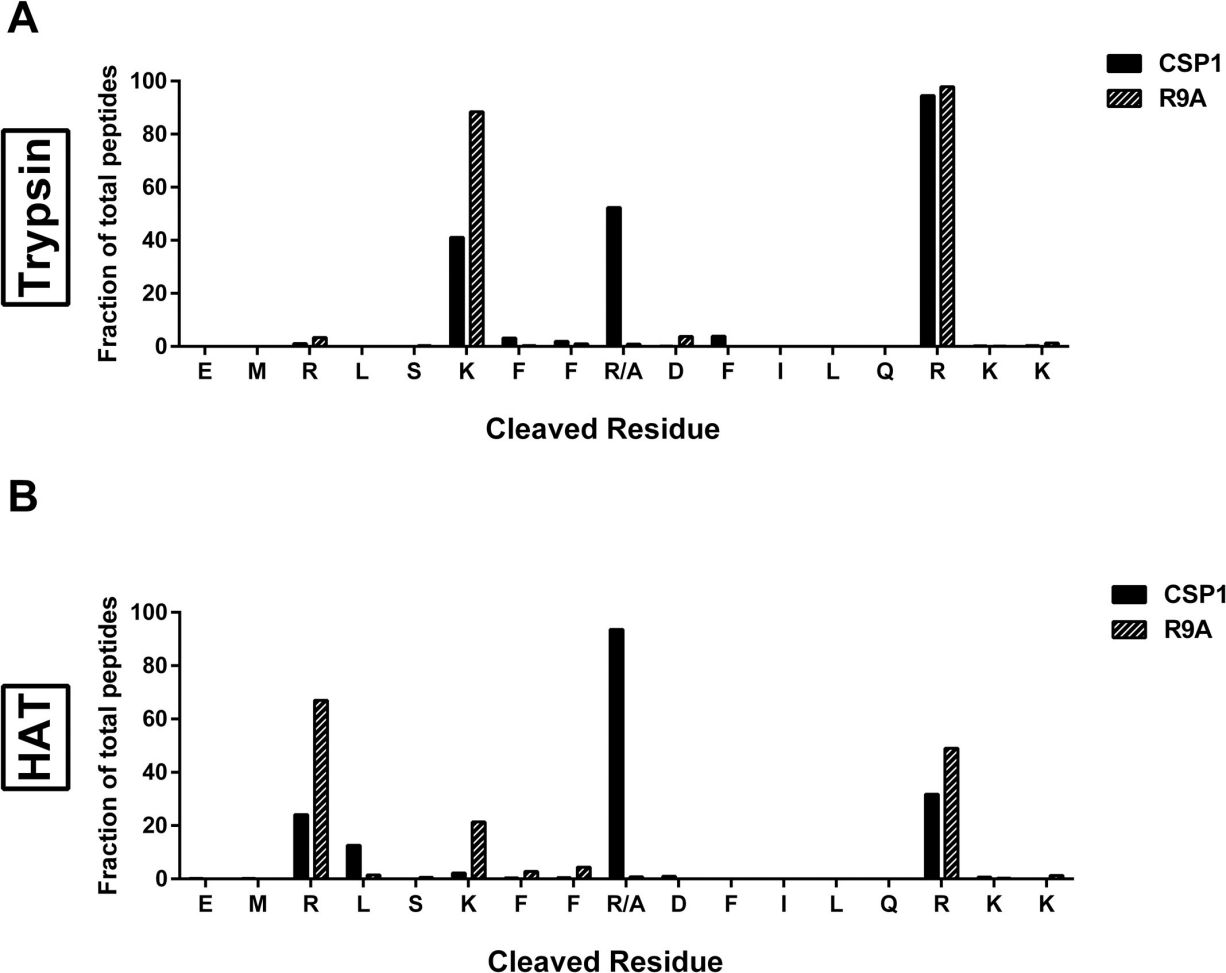
Modification of R9 of CSP1 alters protease digestion profile. Mass spectrometry analysis of cleaved residues of CSP1 (black) or CSP1 R9A (striped) upon incubation with **(A)** porcine trypsin or with **(B)** HAT. The fraction of each peptide detected by mass spectrometry was calculated by dividing the area of the cleaved peptide by the total area of all peptides. In the case of two cleavage sites on the same peptide, the fraction was equally attributed to both cleavage sites. Each bar represents the fraction of identified peptides that contained a cleavage event after the amino acid depicted under the bar. Bars above the final amino acid represent uncleaved CSP1.

As PPT and HAT did not cleave equally at all potential cleavage sites (Fig 1), we investigated whether cleavage at some cleavage sites may have more impact on competence than others. Modified CSPs were synthesized that have altered cleavage sites at R3, K6, R9, and R15 whereby the trypsin-like proteases should no longer recognize the site for cleavage. To ensure that the modified CSP could still induce competence, the recombination frequency of D39x *comC*^-^ transformed with these modified CSP was assessed. There was no or slight reduction in recombination frequency for K6H, R9H, and R15H (Fig 4A). However, modification of R3 resulted in complete loss of competence, as previously observed [43]. To ascertain if modification of CSP alters the impact of trypsin-like protease on competence, the recombination frequency of D39x *comC*^-^ transformed with CSP1 or its modified variants incubated with PPT (Figs 4B and S2) or HAT (Figs 4C and S2) was determined. For both PPT and HAT, incubation of K6H and R15H with trypsin-like proteases reduced recombination frequency similarly to that of unmodified CSP1. Incubation of R9H with trypsin-like proteases reduced recombination frequency of D39x *comC*^-^ to a greater extent than the unmodified CSP1—in that lower concentrations of PPT or HAT were sufficient to reduce recombination frequency. For PPT, the concentration to reduce the recombination frequency to below 5% of 0 trypsin was 5 times less compared to unmodified CSP1 (0.01 vs 0.05 μg/mL). For HAT, the concentration was 2 times less compared to unmodified CSP1 (0.5 vs 1 μg/mL). To determine if the profile of cleavage sites was altered upon R9 modification, the modified CSP was incubated with serine-like proteases and the peptide profile was analyzed via mass spectrometry. Upon cleavage with PPT, the proportion of cleaved peptides after K6 increased, while cleavage after R15 remained the same (Fig 5A). Digestion with HAT shifted the cleavage site from primarily R9 to cleave mainly at R3, followed by R15 (Fig 5B). Taken together, these data suggest that different trypsin-like serine proteases have diverse preferential cleavage sites and the cleavage profile can alter recombination frequency.

### Mouse serine proteases reduce *S*. *pneumoniae* competence

**Fig 6:**
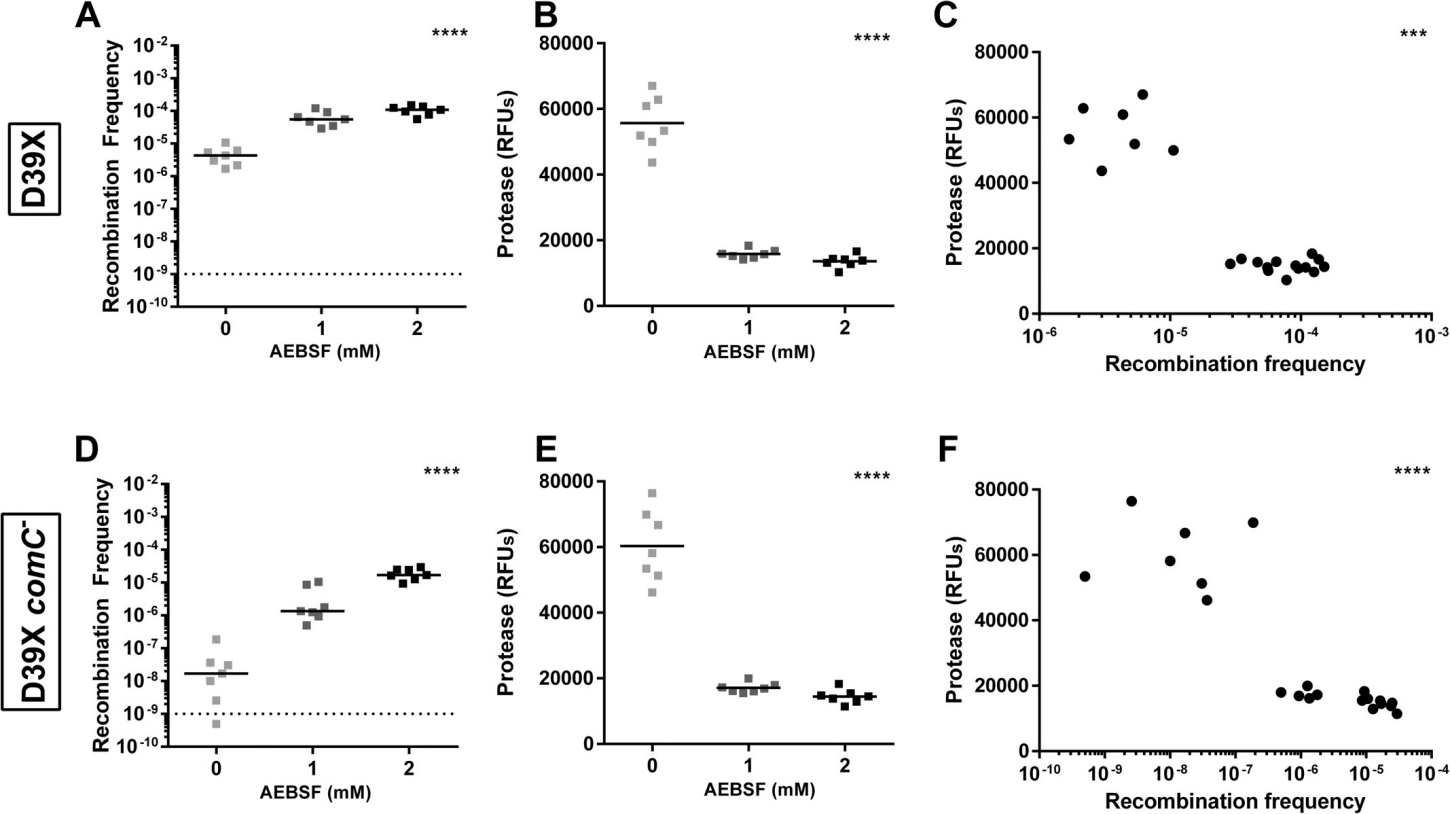
Inhibition of proteases from mouse lungs increase recombination frequency of *S*. *pneumoniae*. Recombination frequency and protease levels upon incubation of CSP1 with homogenized mouse lungs with increasing concentrations of inhibitor AEBSF. **(A)** Recombination frequency of D39x transformed with CSP1 incubated with homogenized mouse lungs. **(B)** Protease levels in homogenized mouse lungs upon incubation with AEBSF used in D39x transformation; determined by fluorescence of substrate t-Butyloxycarbonyl Phe-Ser-Arg 7-amino-4methyl coumarin (BOC). **(C)** Correlation of recombination frequency of D39x with the protease levels in the same homogenized mouse lung. **(D)** Recombination frequency of D39x *comC*^-^ transformed with CSP1 incubated with homogenized mouse lung. **(E)** Protease levels in homogenized mouse lungs upon incubation with AEBSF used in D39x *comC*^-^ transformations; determined by fluorescence of substrate BOC. **(F)** Correlation of recombination frequency of D39x *comC*^-^ with the protease levels in the same homogenized mouse lung. **(A**,**D)** Line represents median; dotted line represents lowest point of detection; recombination frequency of 1 mM and 2 mM AEBSF were compared to 0 mM AEBSF using Kruskal-Wallis one-way ANOVA; ****p<0.0001. **(B**,**E**) Lines represent mean; protease levels of 1 mM and 2 mM AEBSF were compared to 0 mM AEBSF using one-way ANOVA. **(C**,**F)** Correlation was compared using two-tailed spearman; *** p = 0.0004, **** p<0.0001.

To assess if the reduced recombination frequency upon incubation of CSP1 with recombinant trypsin-like proteases could be recapitulated with native airway serine proteases, we determined the recombination frequency of *S*. *pneumoniae* upon incubation of CSP1 with mouse lungs *ex vivo* (Fig 6). Lung homogenates were incubated with increasing concentrations of AEBSF to inhibit native serine proteases prior to incubation with CSP1. Incubation of the lung homogenates with AEBSF increased the recombination frequency of D39x (Fig 6A). This result suggests that the proteases in the lung homogenates reduced recombination frequency and that inhibition of these proteases allowed for increased recombination. Indeed, levels of serine proteases in the lung homogenates were decreased upon incubation with AEBSF (Fig 6B) and there was a strong correlation between the recombination frequency and the level of protease (Fig 6C). A similar result was observed when D39x *comC*^-^ was transformed with CSP1 incubated with lung homogenates (Fig 6D–6F). Of note, AEBSF itself did not increase recombination frequency (S3 Fig).

**Fig 7:**
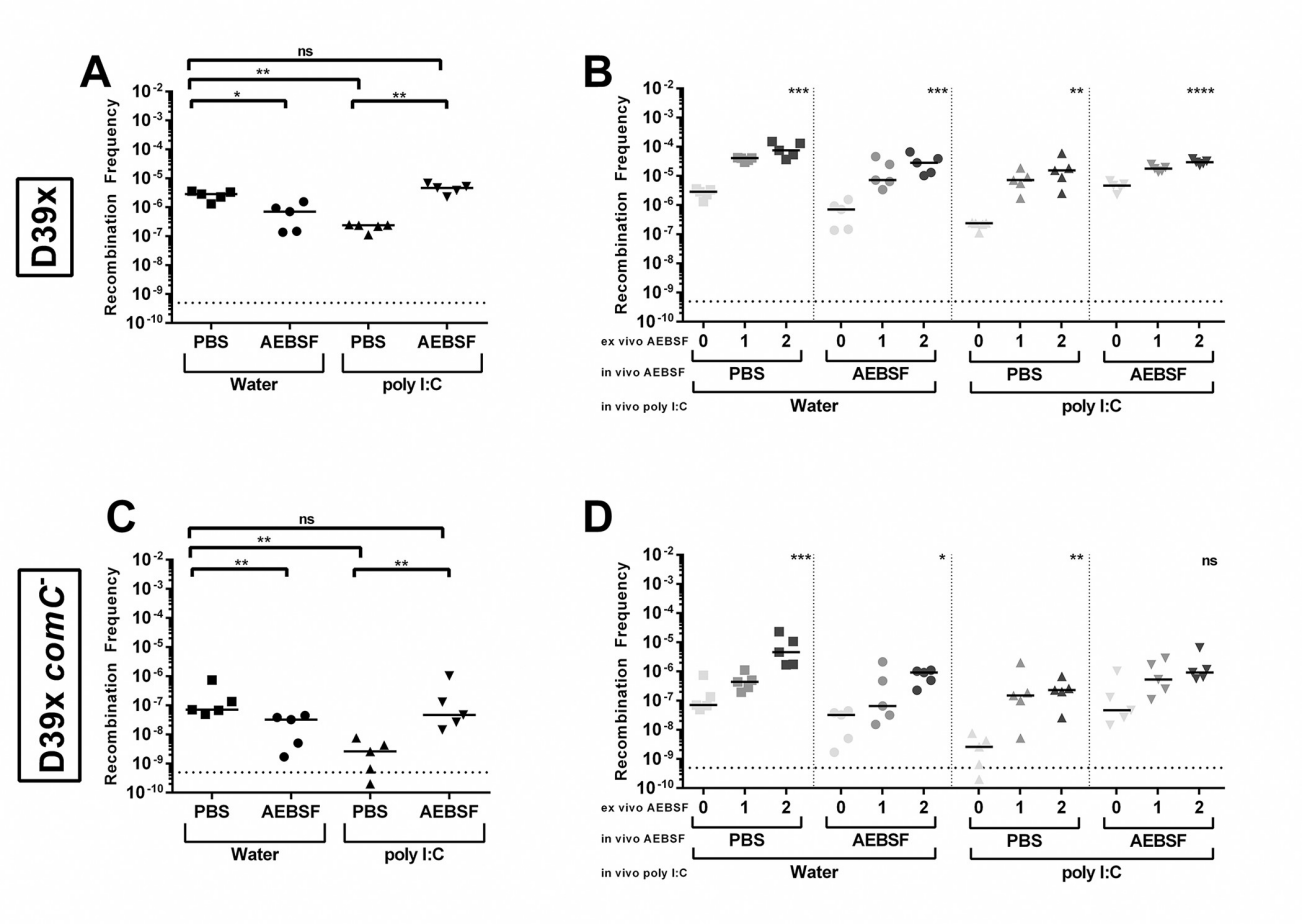
Stimulation of proteases *in vivo* reduces *ex vivo* recombination frequency of *S*. *pneumoniae*. Recombination frequency upon incubation of CSP1 with homogenized lungs from mice that were administered poly (I:C) *in vivo*, treated with inhibitor AEBSF *in vivo*, and then treated *ex vivo* with increasing concentrations of inhibitor AEBSF. **(A)** Recombination frequency of D39x transformed with CSP1 incubated with homogenized lungs from mice that received no stimulant (water) or poly (I:C), and either received no inhibitor (PBS) or inhibitor AEBSF. **(B)** The same lungs were then treated with either 0, 1, or 2 mM AEBSF *ex vivo* prior to incubation with CSP1 and recombination frequency was determined. The 0 mM AEBSF are the same data used in Fig 7A and were included here for comparison. **(C)** Recombination frequency of D39x *comC*^*-*^ transformed with CSP1 incubated with homogenized lungs from mice that received no stimulant (water) or poly (I:C), and either received no inhibitor (PBS) or inhibitor AEBSF. **(D)** The same lungs were then treated with either 0, 1, or 2 mM AEBSF *ex vivo* prior to incubation with CSP1 and recombination frequency was determined. The 0 mM AEBSF are the same data used in Fig 7C and were included here for comparison. Line represents median; dotted line represents lowest point of detection. (**A**,**C**) Recombination frequencies were compared pairwise using nonparametric Mann-Whitney t test; *p = 0.05–0.01, **p = 0.01–0.001. (**B**,**D**) Recombination frequency of 1 mM and 2 mM AEBSF were compared to 0 mM AEBSF of each group using Kruskal-Wallis one-way ANOVA; *p = 0.05–0.01, **p = 0.01–0.001, ***p = 0.001–0.0001, ****p<0.0001.

To confirm that the proteases from mouse lungs could reduce *S*. *pneumoniae* competence, we repeated the *ex vivo* recombination frequency using lungs from mice that were administered poly (I:C) and AEBSF *in vivo*. Poly (I:C) is a synthetic analog of double-stranded RNA that simulates a viral infection and induces the inflammatory response via TLR3 signaling and upregulates serine protease activities [44,45]. Lungs were harvested from mice that were administered poly (I:C) and AEBSF or the corresponding controls intranasally. The homogenized lungs were then incubated with 0, 1, or 2 mM AEBSF, incubated with CSP1, and the lung-AEBSF-CSP mixture was used to transform D39x and D39x *comC*^-^. D39x recombination frequency was reduced when incubated with the homogenized lungs of mice that were previously administered the stimulant poly (I:C) compared to that of control mice. The frequency was restored when the poly (I:C) stimulated mice were administered the protease inhibitor AEBSF IN along with the poly (I:C) (Fig 7A). For all groups, transformation with lungs from mice that were incubated with AEBSF *ex vivo* increased the recombination frequency compared to the lungs homogenates that were incubated with 0mM AEBSF (Fig 7B). This result was similarly observed in the recombination frequency of D39x *comC*^-^ (Fig 7C and 7D). These data further indicate that the proteases in the murine lung can reduce *S*. *pneumoniae* recombination frequency.

**Fig 8:**
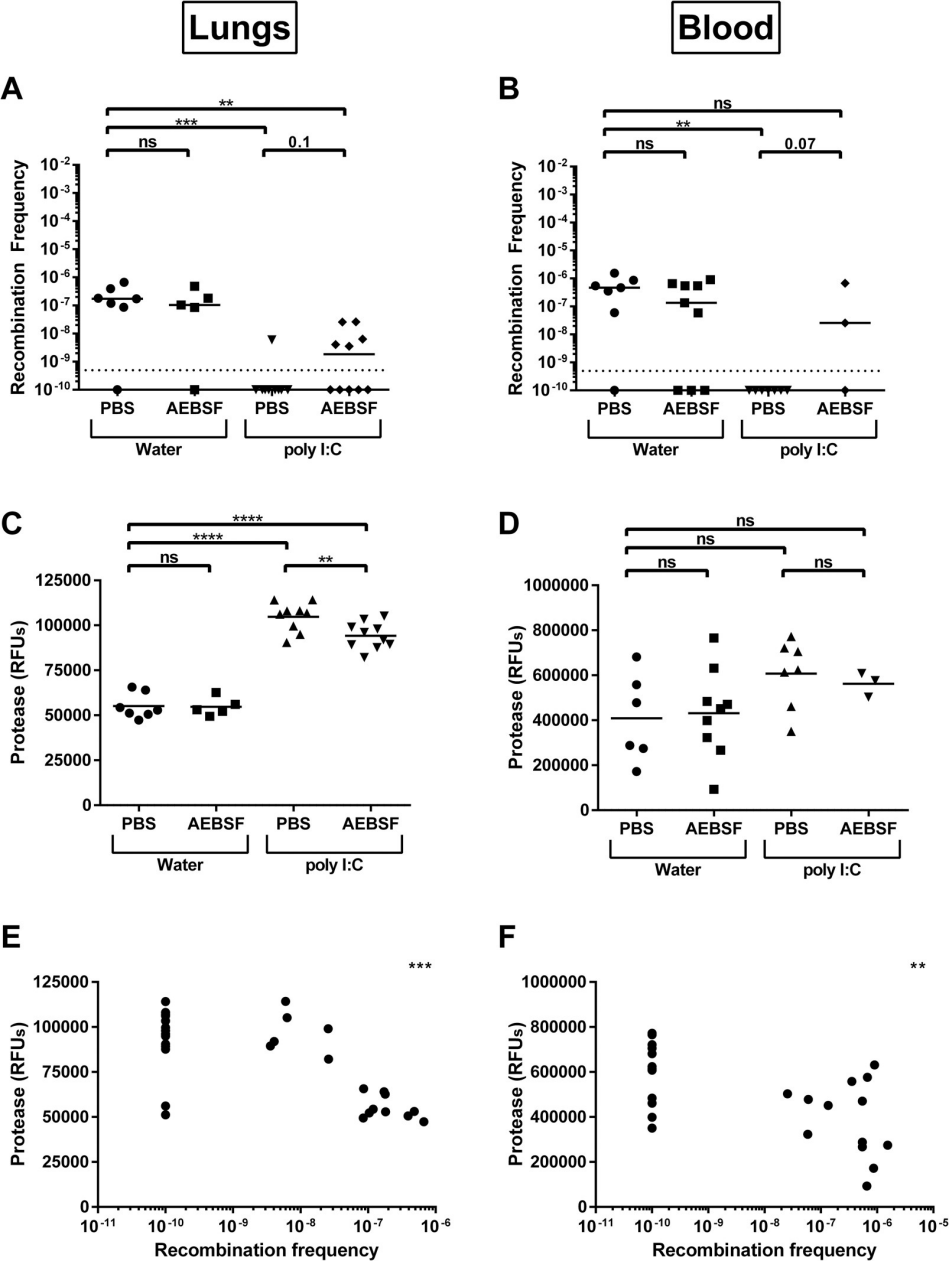
Stimulation of protease production *in vivo* reduces recombination frequency of *S*. *pneumoniae*. Recombination frequency of *S*. *pneumoniae* from **(A)** the lungs and **(B)** the blood of mice that received no stimulant (water) or poly (I:C), and either received no inhibitor (PBS) or inhibitor AEBSF. Protease levels in the same mouse lungs **(C)** and blood (sera) **(D)**; determined by fluorescence of substrate BOC. Correlation of recombination frequency in lungs **(E)** and blood **(F)** with the protease levels in the same tissue. **(A,B)** Line represents median; dotted line represents lowest point of detection; recombination frequencies of all groups were compared pairwise for each tissue using nonparametric Mann-Whitney t test; **p = 0.01–0.001, ***p = 0.001–0.0001. **(C**,**D**) Lines represent mean; protease levels of all groups were compared pairwise for each tissue using unpaired t test; **p = 0.01–0.001, ****p<0.0001. **(E**,**F)** Correlation was compared using two-tailed spearman; **p = 0.004, ***p = 0.0001.

To further investigate if host serine proteases could reduce *S*. *pneumoniae* competence *in vivo*, mice were infected with two strains of D39 that each harbor a different resistance cassette. If competence is induced, the strains would become resistant to both antibiotics upon recombination. To stimulate production of serine proteases in the host, mice were administered inflammatory stimulant, poly (I:C), and serine protease inhibitor, AEBSF, prior to bacterial challenge. A significant reduction in the recombination frequency of D39 in both the lungs and blood of mice administered poly (I:C) was observed compared to the vehicle control (Fig 8A and 8B). Protease levels in the lungs and sera from mice administered poly (I:C) were higher than those from the control mice (Fig 8C and 8D). In mice that were treated with the AEBSF inhibitor prior to poly (I:C) stimulation, recombination occurred at a higher frequency than in mice that were administered poly (I:C) but did not receive the inhibitor (Fig 8A and 8B) but was not completely restored to the frequency observed in mice not administered poly (I:C) (Fig 8A and 8B). The protease levels were reduced in the lungs from mice that were treated with the AEBSF inhibitor prior to poly (I:C) stimulation compared to the lungs from mice that were administered poly (I:C) but did not receive the inhibitor (Fig 8C and 8D). However, this reduced level of protease was still greatly elevated compared to the levels in the lungs from the mice that did not receive poly (I:C). This likely explains why the recombination frequency in the mice administered AEBSF inhibitor prior to poly (I:C) stimulation was not restored to the same frequency of the control mice. The incomplete rescue of the AEBSF inhibitor of the poly (I:C) stimulation is likely an artifact of the *in vivo* model used as the poly (I:C) greatly increases the proteases levels and the dosage and *in vivo* pharmacokinetics of the inhibitor is not sufficient in this model to reduce the levels to baseline. Overall, there was a significant correlation between the recombination frequency and the level of protease in the lungs and blood (Fig 8E and 8F). These data suggest that the native mouse proteases in the lung and blood can reduce *S*. *pneumoniae* recombination frequency *in vivo*.

**Fig 9:**
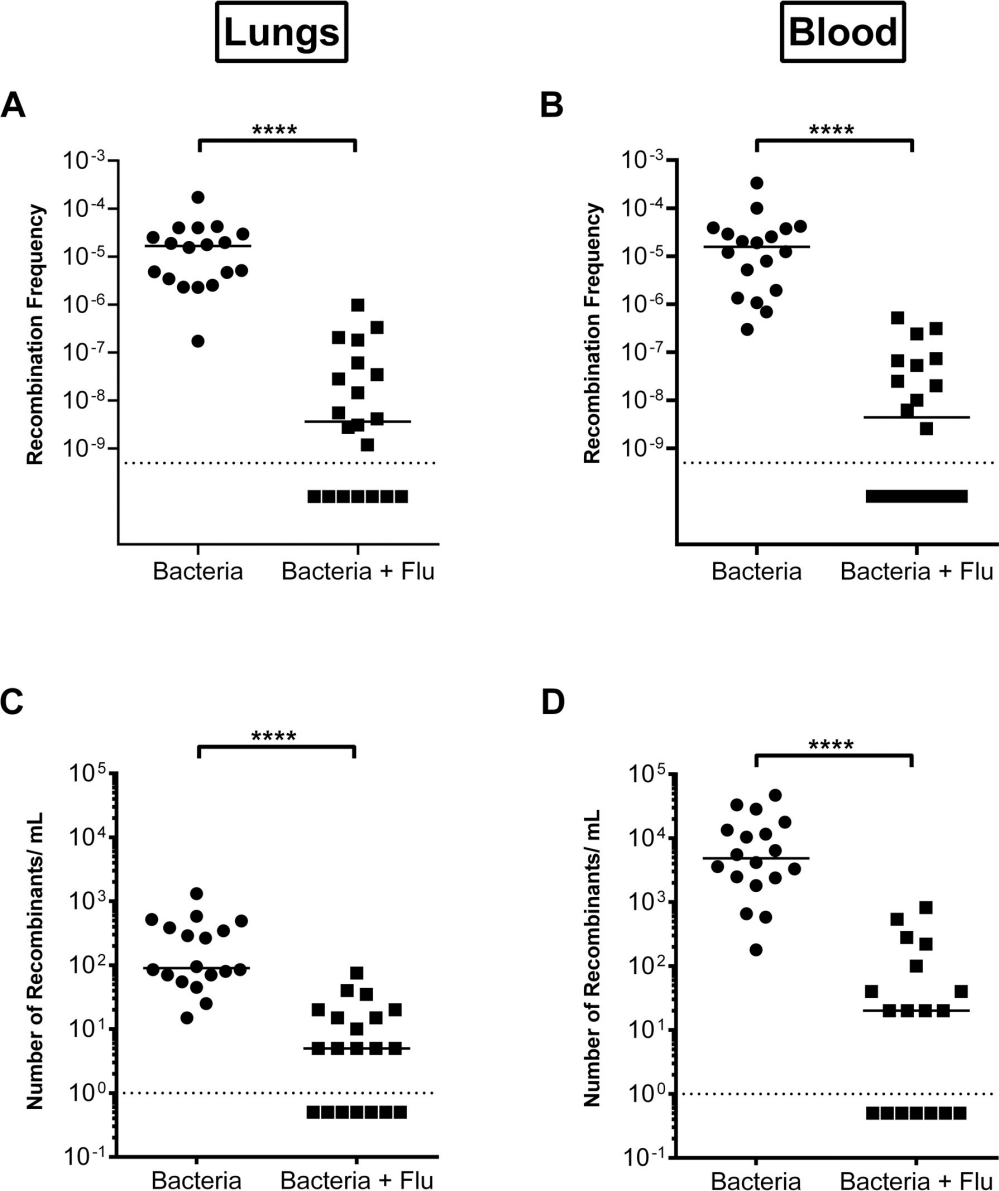
Co-infection with influenza *in vivo* reduces recombination frequency of *S*. *pneumoniae*. Recombination frequency of *S*. *pneumoniae* from **(A)** the lungs and **(B)** the blood of mice infected with and without influenza (Flu). The total number of recombinant colonies per mL used to calculate recombination frequency enumerated from **(C)** the lungs and **(D)** the blood of mice infected with and without influenza (Flu). Lines represent median. Dotted line represents lowest point of detection. Recombination frequency and total number of recombinants from mice infected with influenza was compared to that without influenza for each tissue using nonparametric Mann-Whitney t test; **** p<0.0001.

Influenza virus infection has been shown to induce inflammation and protease production in the respiratory tract and can synergize with *S*. *pneumoniae* secondary infections resulting in exacerbated morbidity and mortality [46–50]. To investigate how coinfection could impact *S*. *pneumoniae* recombination frequency, mice were infected with influenza virus A prior to bacterial challenge. A significant reduction in the recombination frequency of D39 in both the lungs and blood of flu-infected mice was observed (Fig 9), further substantiating the results observed with the poly (I:C) stimulation using a live viral challenge. Taken together, these results indicate that mouse serine proteases can reduce *S*. *pneumoniae* competence.

## Discussion

The innate ability of *S*. *pneumoniae* to recombine exogenous DNA provides a powerful mechanism to adapt to the host environment and this is dependent on CSP [25]. In this study, we have demonstrated that host serine proteases can degrade the pneumococcal competence stimulating peptide, CSP. This is supported by previous findings that demonstrate crude cell-free extracts were sensitive to trypsin digestion [20]. We also show that this degradation has a direct impact on pneumococcal recombination frequency. While the competence cascade has been studied extensively, pneumococcal competence is a complex community trait and the exact triggers and modulators for competence in the mammalian host is not as extensively delineated. As such, advancing our understanding of host and environmental factors that modulate competence remains an important area of investigation.

As a human pathogen, it is possible that the pneumococcus has evolutionarily adapted to exploit the degradation of CSP by the host serine proteases in a manner to thrive in the host. This could explain why the pneumococcus would maintain such an advantageous peptide to be sensitive to proteolytic digestion of the host. As demonstrated here, the CSP sites sensitive to protease degradation can be altered without significant loss of competence, with the notable exception of R3. Many pathogens are known to hijack host serine proteases to promote pathogenesis, including viruses such as influenza and SARS-CoV coronavirus and bacteria such as *Neisseria meningitidis* and *Staphylococcus aureus* [11,51–53]. In particular, influenza virus has been shown to target the human airway trypsin-like protease [54], which we show here degrades CSP and reduces pneumococcal competence. Previous studies have demonstrated that *S*. *pneumoniae* is capable of responding to other host molecules such as LL-37 [55] and of exploiting host proteases, including plasmin and enolase, to promote adherence to epithelial cells of the respiratory tract and to facilitate colonization and invasion [56,57].

**Fig 10:**
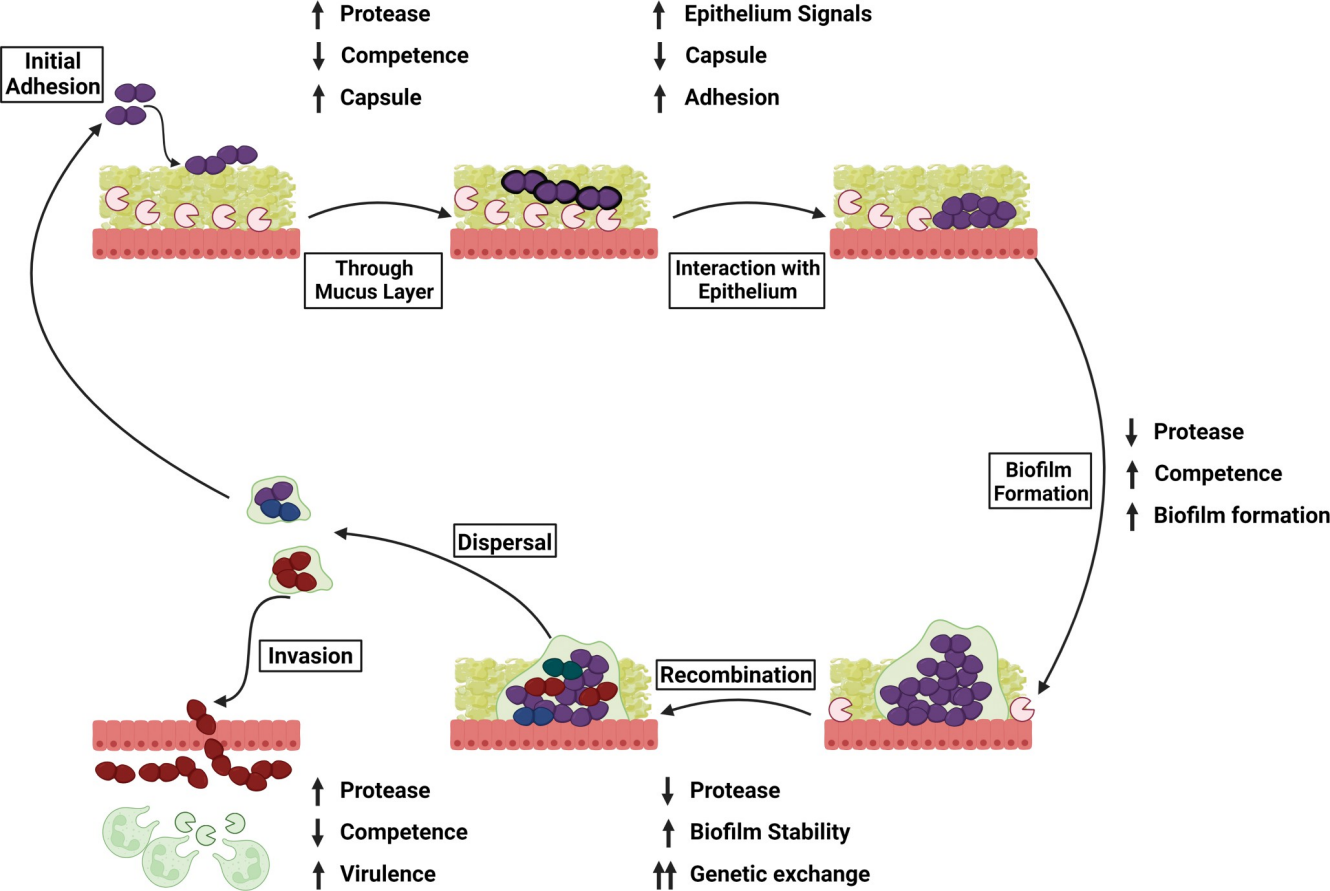
Proposed model of impact of host serine proteases on *S*. *pneumoniae* adaptation to host epithelium and invasion. Created with BioRender.com.

Based on the observations from this study and on previous studies, we propose a framework whereby *S*. *pneumoniae* manipulates the host serine protease degradation of CSP to adapt to the dynamic host environment (Fig 10). When the pneumococcus first encounters the nasopharyngeal epithelial cells, it interacts with multiple host factors during initial colonization. In this study, we were unable to determine the recombination frequency in the nasopharynx of mice as the total number of bacteria recovered from the nasopharyngeal tissue was too low to detect recombinants despite repeated attempts. This is in contrast to previous studies demonstrating efficient recombination during murine colonization, which may be partially explained by differences in the experimental design and resistance genes being modeled between different studies [32]. We utilized a murine model which focuses on pneumococcal infectivity in the lower respiratory tract as it permitted the use of poly (I:C) stimulant and AEBSF inhibitor, which have been previously investigated in an influenza challenge in the lung [45,58]. To our knowledge, there is no current model that demonstrates the pharmacokinetics of these two compounds in the upper respiratory tract. While demonstration of the impact of protease on recombination in the lower respiratory pathway only is a limitation of this study, previous studies have investigated the relationship between competence and colonization of the upper respiratory tract [59–64]. Deletion of the response regulator, *comE*, results in reduced colonization and increased capsular production in adult mice [62,63]. Another investigation demonstrated that mutants of *comAB*, *comD*, and *comE* were able to colonize at levels similar to that of the wild-type when inoculated individually in an infant rat model [64]. However, the *comE* mutant outcompetes the wild-type strain in a competition assay for colonization, while the *comAB* mutant demonstrates a reduced ability to colonize unless the competence cascade could be induced with CSP produced by co-infected wild-type cells [64]. It is possible that the differences observed could be related to immune status, bioavailability, or structural differences of the niches in the *in vivo* models used. In the respiratory tract, as the bacterial cells aggregate and cell population increases, CSP is produced. However, the CSP would be accessible to the serine proteases present in the mucosal layer of the epithelium and can be degraded. In the sputum of patients with inflammatory airway diseases, the concentration of HAT varied in the range of 5–40 nM (0.13 to 1.08 μg/mL) for HAT activity and 2–100 nM (0.05 to 2.7 μg/mL) for the HAT antigen [13,14,65]. Previous reports have demonstrated influenza infection results in a significant increase in trypsin levels in murine lungs following challenge, with approximately twice of much protease being detected compared to uninfected controls [50]. In this study, we observed reduced recombination frequency in the murine lung upon stimulation of serine proteases (Fig 8). Using recombinant HAT to generate a standard for the protease assay, we can equate 35000 RFU to be 0.05 μg/mL recombinant HAT and the protease levels to be 0.079 (±0.010) μg/mL in non-inflamed lungs and 0.168 (±0.012) μg/mL in inflamed lungs, which are similar to those observed in the sputum of patients. In the *in vitro* recombination experiment, we observed reduced recombination frequency when CSP was incubated with at least 0.5 μg/mL HAT in D39x *comC*^-^ and with at least 10 μg/mL HAT in D39x (Fig 2). The higher concentrations required to reduced recombination frequency *in vitro* compared to those observed in human sputum/ murine lungs could be reflective of differences in the HAT protease itself (recombinant vs native), the concentration of CSP used *in vitro* compared to those found in the host, the likely factor of additional host serine proteases that may also contribute to the observed levels of recombination frequency *in vivo*, and the potential variable distribution of proteolytic activity across the lung. These important caveats should be taken into consideration in the interpretation of the experimental data.

In the host, the serine proteases could reduce levels of CSP and, as the threshold required to stimulate the competence cascade is not met [66,67], competence would be reduced. This could promote passage through the mucus layer via reduced capsule or dysregulation of other factors, allowing access to the epithelium [68,69]. Once through the mucus layer, interaction with the epithelium stimulates capsular shedding which can tighten adherence [55,70,71] and potentially enhances competence [72]. Cell density increases and biofilm formation is upregulated [73,74]. The biofilm protects the growing population from host immune defenses (such as proteases) [75,76] and pneumococcal cells adapt to their host environment, downregulating factors that can stimulate inflammation [77]. This loss of pressure from host serine proteases combined with the proximity of cells within the biofilm provides a golden opportunity for the pneumococcus to increase competence for genetic exchange to best survive in the host environment and increase fitness [31,32]. As the biofilm matures, host-adapted cells can be triggered by host signals to disperse and either reattach and begin the colonization process anew or invade. These host-adapted cells dispersed from the biofilm are more inflammatory and invasive since they disseminate into lungs/ middle ear more readily than either broth-grown bacteria or bacteria that are prematurely dispersed from a biofilm [74,78,79]. The dispersed cells have also been shown to downregulate genes involved in competence and colonization [80]. Inflammation induced by invasive cells promotes neutrophil recruitment and proteases production, which could further reduce pneumococcal competence. A similar model may be applied to the lower respiratory tract, where the production and response to CSP is required for pneumococcal survival and pathogenesis and biofilm-like structures may be present to modulate exposure of CSP to host proteases [26,43,59,81–83]. While biofilm and colonization in the nasopharynx are of central importance for genetic exchange [32,36], the pneumococcus can remain competent in the lungs and blood [84] and, as shown here, recombination frequency can be reduced in the lungs of mice that have been administered stimulant poly (I:C) or co-infection with influenza (Figs 8 and 9). Prolonged colonization by multiple strains likely increases the probability of successful recombination events compared to the acute invasive model used in this study to interrogate recombination frequency, which is an important consideration when putting these findings in a biological context. This underscores the importance of understanding pneumococcal recombination events not only in the upper respiratory, but in the lower respiratory as well—in particular, how the pneumococcus can adapt to host and antibiotic pressures during an infection.

Pneumococcal competence is a complex community trait and is likely modulated by a multitude of factors in the mammalian host. In this study, we have demonstrated one mechanism by which the host can modulate recombination and how the pneumococcus may adapt to the host serine proteases. It is also possible that other factors besides host serine proteases impact recombination efficiency in the host, including those that induce oxygen exposure, SOS, and DNA replication stress, but the direct impact of these factors on recombination in the host has not been extensively delineated [41,85,86]. Another key consideration is the growth state of the pneumococcus in these tissues, as the transition from biofilm to planktonic growth can also play an important role in recombination frequency. The pneumococcus also encodes several serine proteases (PrtA, SFP, CbpG, HrtA) that are involved in competence, colonization, lung inflammation, and survival in the blood. In particular, HrtA plays an important role in maintaining redox balances and capsule production upon stimulation of the host inflammatory response, for example during an influenza co-infection [87–93]. It is unclear how these bacterial proteases interact, if at all, with host airway proteases or proteases encoded by co-colonizing bacterial species. Nonetheless, these findings underscore the importance of host proteases in the modulation of pneumococcal competence and the complexity of the multi-niche interaction between *S*. *pneumoniae* and the host. These data indicate that host proteases can modulate pneumococcal competence through the degradation of a microbial signaling peptide by host proteases, suggesting conditions whereby repression or upregulation of such proteases may impact pneumococcal genetic exchange in opposing ways, underscoring the importance of the host environment on this critical facet of pneumococcal biology.

## Material and methods

### Ethics statement

All experiments involving animals were performed with prior approval of and in accordance with guidelines of the St. Jude Institutional Animal Care and Use Committee. The St Jude laboratory animal facilities have been fully accredited by the American Association for Accreditation of Laboratory Animal Care. Laboratory animals were maintained in accordance with the applicable portions of the Animal Welfare Act and the guidelines. All mice were maintained in ABSL2 facilities and all experiments were done while the mice were under inhaled isoflurane (2.5%) anesthesia.

### Media and growth conditions

*Streptococcus pneumoniae* was grown on tryptic soy agar (EMD Chemicals, NJ, USA) supplemented with 3% defibrinated sheep blood and 20 μg/mL neomycin (TSA blood plates). Cultures were inoculated from newly streaked TSA blood plates into C+Y, a semi-synthetic casein liquid media with 0.5% yeast extract [94] and grown at 37°C, 5% CO_2_. Strains used in this study are listed in Table 1.

**Table 1:** Strains used in this study.

Strain	Description	Source
D39x	Bioluminescent D39 with Tn4001 *luxABCDE*; Kan^R^	Francis, et al [95]
D39x *comC*^-^	Replacement of *comC* (Spd_2065) with PhunSweetErm cassette in D39x; Kan^R^ Erm^R^	This study
TIGR4	TIGR4 wild-type strain	
TIGR4 *comC*^-^	Replacement of *comC* (Sp_2237) with PhunSweetErm cassette in TIGR4; Erm^R^	This study
DLA3	D39 with luciferase under the promoter of *ssbB* (P*ssbB-luc)*	Slager, et al [41]
D39 Tn-Seq	Tn-Seq library generated in D39; Spec^R^	Matthews, et al [96]
TIGR4Δcps::PhunSweet	Source of PhunSweet cassette	Echlin, et al [97]
TIGR4 Tn-Seq	Tn-Seq library generated in TIGR4; Spec^R^	Van Opijnen, et al [98]
TIGR4ΔspxB::Erm	Source of *erm* cassette	Echlin, et al [99]

### Generation of *comC*^-^ deletion mutant

To delete *comC*, Spd_2065 was replaced with a modified PhunSweet cassette [97] via SOE PCR [100]. All PCR products were amplified using exTaq polymerase (TAKARA) following the recommended guidelines and primers are listed in Table 2. gDNA was extracted using the aqueous/organic extraction protocol, as described previously [97]. To utilize the PhunSweet cassette in the D39x background (which has kanamycin resistance), the kanamycin resistance *kan* in the PhunSweet cassette was replaced with erythromycin resistance *erm*, generating PhunSweetErm. The PhunSweet cassette (minus *kan*) was amplified from TIGR4Δcps::PhunSweet gDNA using primers PhunSweet_F and PhunSweet-kan_R. The erythromycin resistance cassette *erm* was amplified from TIGR4ΔspxB::Erm [99] gDNA using primers Erm_F and Erm_R. PhunSweet-kan and Erm PCR products were spliced together using PhunSweet_F and Erm_R primers. To generate the *comC*^-^ deletion mutant, 1 kb fragments upstream and downstream of *comC* were amplified from D39 gDNA using primers ComC_Up_F/ ComC_Up_R and ComC_Down_F/ ComC_Down_R, respectively. These amplicons were spliced with the PhunSweetErm cassette with primers ComC_Up_F and ComC_Down_R to generate *comC*::PhunSweetErm. For generation of D39x *comC*^*-*^, D39x was grown in 10 mL C+Y until OD_620_~0.08. 1 mL of culture was incubated with 3 μL of 1 mg/ml CSP1 [39] and the *comC*::PhunSweetErm amplicon for three hours at 37°C, 5% CO_2_. Transformants were selected on plates containing 1μg/mL erythromycin. Correct deletion of *comC* was confirmed through lack of growth on counter-selection plates (15mM chlorinated-phenylalanine, 10% sucrose) and through PCR. To generate the *comC* mutant in TIGR4, the *comC*::PhunSweetErm amplicon was generated in the same manner as above using the same primers, but TIGR4 gDNA was the source of DNA. TIGR4 was transformed with the *comC*::PhunSweetErm amplicon following the same protocol as above, except for using CSP2 instead of CSP1.

**Table 2:** Primers used to generate D39x *comC*^-^.

Name	Sequence
PhunSweet_F	CAATTAACTTTACAAATTCCCACTATTAAGG
PhunSweet-kan_R	**GTTTGCTTCTAAGTCTTATTTCC**ACTTTTGTGCCCGTGCTTATAAGGG
Erm_F	GGAAATAAGACTTAGAAGCAAAC
Erm_R	CCAAATTTACAAAAGCGACTC
ComC_Up_F	GAAAAACTACCCAAGGCTCCACT
ComC_UP_R	**ATAGTGGGAATTTGTAAAGTTAATTG**AATAAAATCTCCTAAAATGTTTTTTCTTG
ComC_Down_F	**GAGTCGCTTTTGTAAATTTGG**TGAAATAAGGGGAAAGAGTAATGGATTTAT
ComC_Down_R	TAGCTATCAGCCGATCCTTCG

For PhunSweet-kan_R, ComC_Up_R, and ComC_Down_F, nucleotides in bold are overlapping sequence of 5’ of erm, 5’ of PhunSweet, and 3’ of erm, respectively.

### CSP synthesis

CSP1, CSP2, and all modified variants of CSP1 were synthesized and HPLC purified by the peptide synthesis core at St. Jude Children’s Research Hospital. CSPs were reconstituted in nuclease-free water at a concentration of 1 mg/mL. For CSP1, the amino acid sequence was EMRLSKFFRDFILQRKK; for CSP2, the sequence was EMRISRIILDFLFLRKK; for R3H, the sequence was EMHLSKFFRDFILQRKK; for K6H, the sequence was EMRLSHFFRDFILQRKK; for R9H, the sequence was EMRLSKFFHDFILQRKK; for R9A, the sequence was EMRLSKFFADFILQRKK; for R15H, the sequence was EMRLSKFFRDFILQHKK.

### Mass spectrometry

CSPs was incubated with either porcine trypsin gold (Promega #V5280) or human airway trypsin-like protease (HAT; Bio-techne R&D #2695-SE-010) for thirty minutes, room temperature. After thirty minutes, 10% acetic acid was added to stop further digestion. Peptide samples were loaded on a nanoscale capillary reverse phase C18 column by a HPLC system (Thermo Ultimate3000) and eluted by a gradient (~30 min). The eluted peptides were ionized by electrospray ionization and detected by an inline mass spectrometer (Thermo Orbitrap Fusion). High resolution MS1 spectra were collected in the orbitrap and followed by MS/MS of the 20 most abundant ions using the ion trap. This process was cycled over the entire liquid chromatography gradient. Database searches were performed using Sequest search engine in an in-house SPIDERS software package. All matched MS/MS spectra were filtered by mass accuracy and matching scores to reduce protein false discovery rate to ~1%. The total number of spectra, namely spectral counts (SC), matching to individual proteins may reflect their relative abundance in one sample after the protein size is normalized. Fractional area of each peptide and calculations to generate figures are included as S1 File.

### *in vitro* recombination frequency

Strains were inoculated in C+Y at OD_620_~0.03. During culture incubation, porcine pancreatic trypsin (PPT; Sigma #T4799) was reconstituted in Hank’s balanced salt solution (Gibco) and incubated with either water or 4-(2-Aminoethyl) benzenesulfonyl fluoride hydrochloride (AEBSF; Cayman #14321) in a total volume of 50μL 30mM Tris, pH 8.5 and incubated for 15 minutes, room temperature. CSP1 or CSP2 was added, followed by incubation for 30 minutes, room temperature. When the culture reached OD_620_~0.08, 1 mL of culture was transferred to the PPT-AEBSF-CSP mixture and 2 μg of D39 Tn-seq library gDNA (D39 strains) or 2 μg of TIGR4 Tn-seq library gDNA (TIGR4 strains) was added. For D39x *comC*^*-*^, TIGR4, and TIGR4 *comC*^*-*^, the culture was pelleted at 6,000xg, 5 min and resuspended in fresh C+Y (to increase recombination efficiency; S3 Fig) before the culture was transferred to the mixture. Upon addition of the culture, the final concentration of PPT ranged from 0–10 μg/mL, AEBSF was 0.1 mM, CSP was 3 μg/mL, and gDNA was 2 μg/mL. The transformation was incubated for 3 hours at 37°C, 5% CO_2_, followed by serial dilution and plating on two TSA blood plates and two TSA blood plates supplemented with 200 μg/mL spectinomycin. To observe low-frequency recombination events, 200 μL of the transformation was also spread plated on two TSA blood plates supplemented with 200 μg/mL spectinomycin. For transformations with HAT incubated CSP, HAT (Bio-techne R&D #2695-SE-010) was reconstituted in 25mM Tris, 150mM NaCl, pH 7.5. All incubation times and final concentrations were the same as for PPT; however, the volumes were 1/10^th^ as that for PPT. For both PPT and HAT, the experiment was repeated at least three times. Recombination frequencies in increasing concentrations of protease within each group (0 mM AEBSF or 0.1 mM AEBSF) were compared using Kruskal-Wallis one-way ANOVA.

### Luminescence assay

To further investigate the impact of trypsin on competence, changes in luminescence upon incubation of CSP with trypsin in the strain DLA3, generously provided by Jan-Willem Veening [41], were monitored. A similar protocol as the *in vitro* recombination frequency was followed. DLA3 was inoculated in C+Y at OD_620_~0.03. Either PPT or HAT was incubated with water or AEBSF for 15 minutes, room temperature, followed by incubation with CSP1 for thirty minutes in a total volume of 5 μL 30mM Tris, pH 8.5. When the culture reached OD_620_~0.08, cells were pelleted at 6,000xg, 5 min and resuspended in fresh C+Y. 100 μL of the culture was added to the Trypsin-AEBSF-CSP mixture, followed by transfer to a white-walled, clear-bottom 96-well plate and addition of 5 μL of 10 mg/mL luciferin (Sigma #L6882). Luminescence and OD_620_ measurements were taken every 30 minutes in a Biotek Cytation 3. Increasing concentrations of protease within each group (0 mM AEBSF or 0.1 mM AEBSF) were compared using two-way ANOVA in Graphpad 6.

### *ex vivo* recombination frequency

To determine the impact of proteases from the lungs of mice on recombination frequency, a similar experimental setup as the *in vitro* recombination frequency was performed. D39x and D39x *comC*^*-*^ were inoculated in C+Y at OD_620_~0.03. During culture incubation, the lungs of 10-week-old female Balb/cJ mice (N = 7) were harvested and homogenized in 600 μL PBS. Lung homogenate was incubated with either water or AEBSF for 30 minutes, room temperature. 60 μL of lung homogenate-AEBSF mixture was transferred to a new tube. Addition of CSP1, culture, and gDNA and transformation was performed in the same manner as the *in vitro* recombination frequency. The final concentration of CSP was 3 μg/mL and gDNA was 2 μg/mL. The final concentration of AEBSF was 0mM, 1 mM, or 2mM. The lung from each mouse was divided into six transformations: D39x with lungs incubated with 0mM, 1 mM, or 2mM AEBSF and D39x *comC*^*-*^ with lungs incubated with 0mM, 1 mM, or 2mM AEBSF. As a control, the same culture of D39x and D39x *comC*^*-*^ were transformed with gDNA and CSP1 incubated with AEBSF in PBS. Recombination frequency of the transformation with CSP1 incubated with 1 mM and with 2 mM AEBSF were compared to that incubated with 0 mM AEBSF using Kruskal-Wallis one-way ANOVA in Graphpad 6. Of note, the concentration of AEBSF was higher here than for the *in vitro* recombination frequency experiments due to potential other components in the lung homogenate reducing the efficacy of the inhibitor.

For *in vivo* treatment of mice prior to *ex vivo* recombination frequency experiment, 7-week-old female Balb/cJ mice were treated with an inflammatory stimulant, poly (I:C), or water control and with inhibitor AEBSF or PBS control. 50 μL of 1 mg/mL poly(I:C) HMW (Invivogen) or 50 μL water control was administered IN daily for four days prior to harvest (N = 10) [45]. In the same mice, either 125 μg/ 25 μL AEBSF or 25 μL PBS control was administered IN daily for three days prior to harvest (N = 5) [58]. Lungs were harvested and homogenized in 600 μL PBS. The homogenates from all lungs were subjected to the *ex vivo* protocol described above and the recombination frequency of D39x and D39x *comC*^*-*^ subjected to lungs incubated with 0mM, 1 mM, or 2mM AEBSF and CSP1 was determined. Recombination frequencies of 0mM AEBSF groups were compared pairwise for each tissue using nonparametric Mann Whitney t test and recombination frequency of 1 mM and 2 mM AEBSF were compared to 0 mM AEBSF within each group using Kruskal-Wallis one-way ANOVA in Graphpad 6.

### Protease levels

For *ex vivo* experiments, the protease levels were measured from the same lung homogenate used in the *ex vivo* recombination frequency experiment, adapting the protocol from the HAT manufacturer (Bio-techne R&D). After the lungs were incubated with either water or AEBSF, 50 μL of lung homogenate mixture was transferred to a black, 96-well plate. 50 μL of substrate t-Butyloxycarbonyl Phe-Ser-Arg 7-amino-4methyl coumarin (Bachem # I1400) was added directly to the well, incubated for 10 minutes at room temperature, and fluorescence (380 excitation; 460 emission) was detected in a Biotek Cytation 3. Protease levels of lung homogenates incubated with 1 mM and 2 mM AEBSF were compared to that incubated with 0 mM AEBSF using one-way ANOVA and correlation between protease levels and recombination frequency was compared using two-tailed spearman in Graphpad 6.

For *in vivo* experiments, homogenized lungs were further clarified after plating on TSA plates via centrifugation at 10,000xg, 5min. Sera was separated from whole blood via centrifugation at 10,000xg, 5 min in sera-collection tubes. The clarified homogenates and sera were transferred to a black, 96-well plate and 50 μL of substrate t-Butyloxycarbonyl Phe-Ser-Arg 7-amino-4methyl coumarin was added directly to the well and fluorescence (380 excitation; 460 emission) was detected in a Biotek Cytation 3. Protease levels of all groups were compared pairwise for each tissue using unpaired t test and correlation between protease levels and recombination frequency was compared using two-tailed spearman in Graphpad 6.

### *in vivo* recombination frequency

To stimulate production of protease *in vivo*, 7-week-old female Balb/cJ mice were treated with an inflammatory stimulant, poly (I:C), or water control and with serine protease inhibitor, AEBSF, or PBS control. 50 μL of 1 mg/mL poly(I:C) HMW (Invivogen) or 50 μL water control was administered IN daily for four days prior to bacterial challenge. In the same mice, either 125 μg AEBSF or PBS control was administered IN daily for two days prior to poly (I:C) treatment, two hours prior to daily poly (I:C) treatment, and two hours prior to bacterial challenge. All mice were challenged with a 90:10 mixture of D39x (kanamycin resistance) and D39 Tn-seq library (spectinomycin resistance) intranasally at 10^7^ CFU in 100 μL. At 20 hours following challenge, lungs and blood were harvested. Lungs were homogenized in PBS and lungs and blood were serially diluted and plated. To detect for recombinants, tissues were plated on TSA blood plates plus 400 μg/mL kanamycin plus 200 μg/mL spectinomycin in addition to plates without antibiotic and plates with either antibiotic alone to quantitate the respective bacterial populations. Plates were incubated overnight at 37°C, 5% CO_2_ and recombinant colonies were enumerated. Any sample that had a total bacterial burden below 10^7^ CFU/mL was excluded from calculated recombination frequency as it falls below the threshold to observe recombinants (S4 Fig). The calculated recombination frequency (number of recombinant colonies divide by total number of colonies) of all groups were compared for each tissue using nonparametric Mann-Whitney t test in Graphpad 6.

To investigate impact of protease induction due to influenza infection [49,50] prior to bacterial challenge *in vivo*, influenza A/California/04/2009 at 5x10^2^ LD_50_ was intranasally administered to 8-week-old female Balb/cJ mice (N = 20). Influenza A/California/04/2009 virus was purified as described previously [101]. As a control, PBS was intranasally administered to 8-week-old female Balb/cJ mice (N = 20). Seven days post-infection, the same mice were infected with a 90:10 mixture of D39x (kanamycin resistance) and D39 Tn-seq library (spectinomycin resistance) intranasally at 10^5^ CFU in 100 μL. At 8 hours post-bacterial challenge, mice were treated with 2.5 mg/kg spectinomycin (adjusted for potency) to drive recombination from the spectinomycin resistant donor strains to the kanamycin resistant, spectinomycin sensitive strains. At 12 hours following spectinomycin treatment, lungs and blood were harvested. Lungs were homogenized in PBS and both blood and lungs were serially diluted and plated. Tissues were plated, recombinant colonies enumerated, and recombination frequency calculated as above. Number of recombinant colonies and the calculated recombination frequency (number of recombinant colonies divide by total number of colonies) detected in mice infected with influenza was compared to that in mice that received PBS for each tissue using nonparametric Mann-Whitney t test in Graphpad 6.

## Supporting information

S1 Fig Growth of *S*. *pneumoniae* is not reduced by addition of AEBSF or trypsin. Absorbance at OD_620_ of DLA3 grown in the presence of CSP1 incubated with increasing concentrations of trypsin with and without inhibitor AEBSF. Incubation of CSP1 with PPT **(A)** without AEBSF or **(B)** with AEBSF. Incubation of CSP1 with HAT **(C)** without AEBSF or **(D)** with AEBSF. Experiment was repeated in triplicate. The mean value of OD_620_ of each 30-minute timepoint is reported; error bars are SEM.
(TIF)

S2 Fig Modified CSP alters impact of protease on recombination frequency. Recombination frequency upon incubation of modified CSP1 with increasing concentrations of serine proteases. Transformation of D39x *comC*^-^ with modified CSP1 incubated with **(A)** PPT or **(B)** HAT. Negative controls included no addition of CSP1 and no addition of gDNA. Lines represent median value. Dotted line represents lowest point of detection. Recombination frequencies of increasing concentrations of protease within each modified CSP were compared using Kruskal-Wallis one-way ANOVA; *p = 0.05–0.01, **p = 0.01–0.001. Changes in the concentrations of protease between modified CSP was compared using two-way ANOVA; all groups non-significant.
(TIF)

S3 Fig AEBSF alone does not induce recombination and D39x *comC*^-^ recombination frequency is increased upon washing with fresh media. **(A)** Recombination frequency of D39x and D39x *comC*^-^ upon incubation of CSP1 in PBS with increasing concentrations of AEBSF. Lines represent median. Dotted line represents lowest point of detection. **(B)** Recombination frequency of D39x *comC*^-^ with and without washing cells with fresh media. Lines represent median. Dotted line represents lowest point of detection.
(TIF)

S4 Fig Total number of cells and recombinants recovered from lungs and blood during *in vivo* recombination frequency experiment. Total number of cells per mL recovered from **(A)** the lungs and **(B)** the blood of mice that received no stimulant (water) or poly (I:C), and either received no inhibitor (PBS) or inhibitor AEBSF. Lines represent median. Dashed line at 10^7^ represents threshold for detection of recombinants. Any bacterial burden that fell below this threshold was excluded from Fig 8. Dotted line represents lowest point of detection. Total number of recombinants per mL recovered from **(C)** the lungs and **(D)** the blood. Lines represent median. Number of recombinants per mL of all groups were compared pairwise for each tissue using nonparametric Mann-Whitney t test; **p = 0.01–0.001.
(TIF)

S1 File Raw mass spectrometry data and calculated proportions for CSP cleavage experiments. (XLSX)
