# Supplementary figures and images for: Airway proteolytic control of pneumococcal competence
Source: PLoS Pathog. 2023 May 31;19(5):e1011421. doi: 10.1371/journal.ppat.1011421 (PMC10259803; doi:10.1371/journal.ppat.1011421)

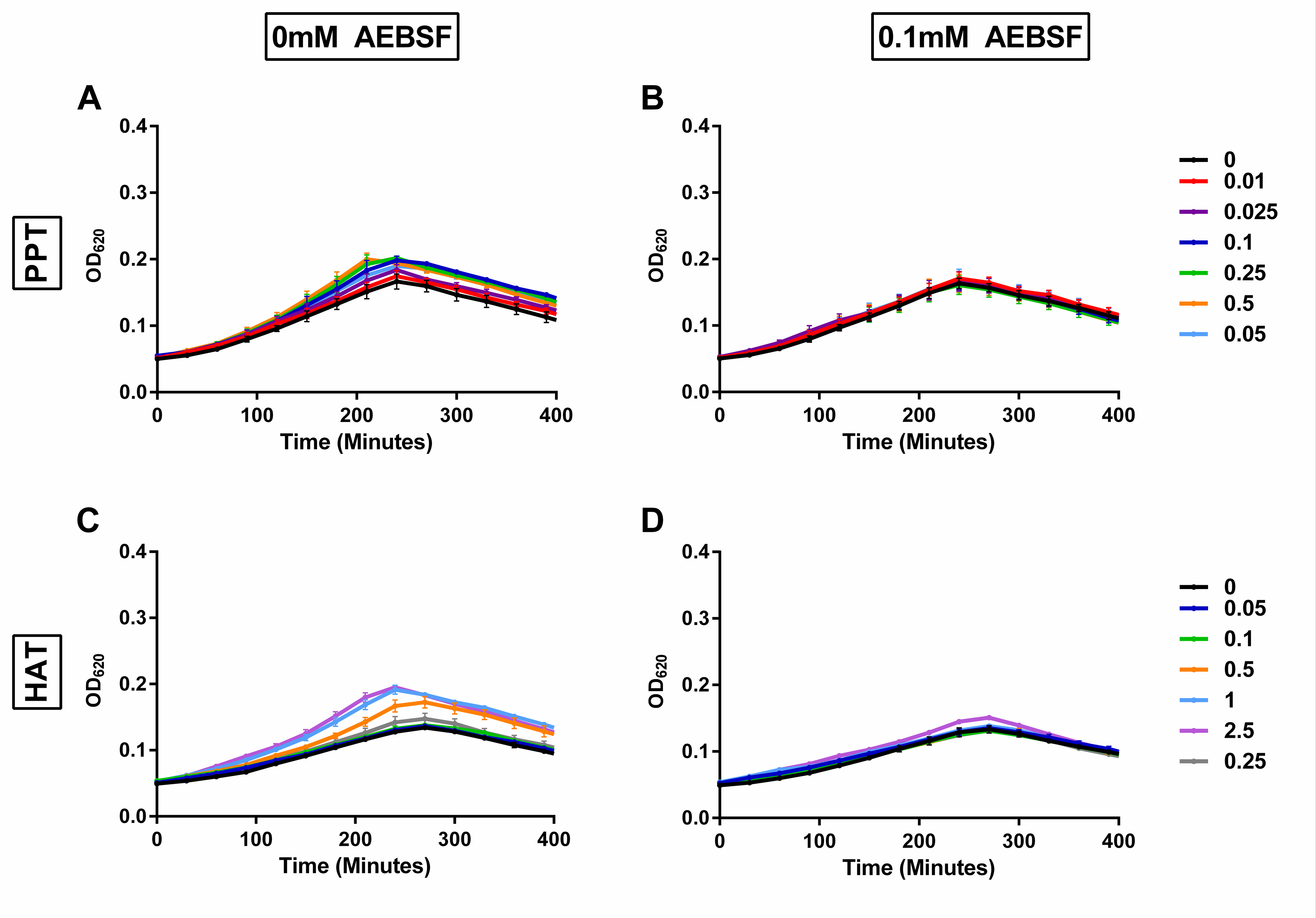

Supplement: S1 Fig — Absorbance at OD620 of DLA3 grown in the presence of CSP1 incubated with increasing concentrations of trypsin with and without inhibitor AEBSF. Incubation of CSP1 with PPT (A) without AEBSF or (B) with AEBSF. Incubation of CSP1 with HAT (C) without AEBSF or (D) with AEBSF. Experiment was repeated in triplicate. The mean value of OD620 of each 30-minute timepoint is reported; error bars are SEM. (TIF) [file ppat.1011421.s001.tif]

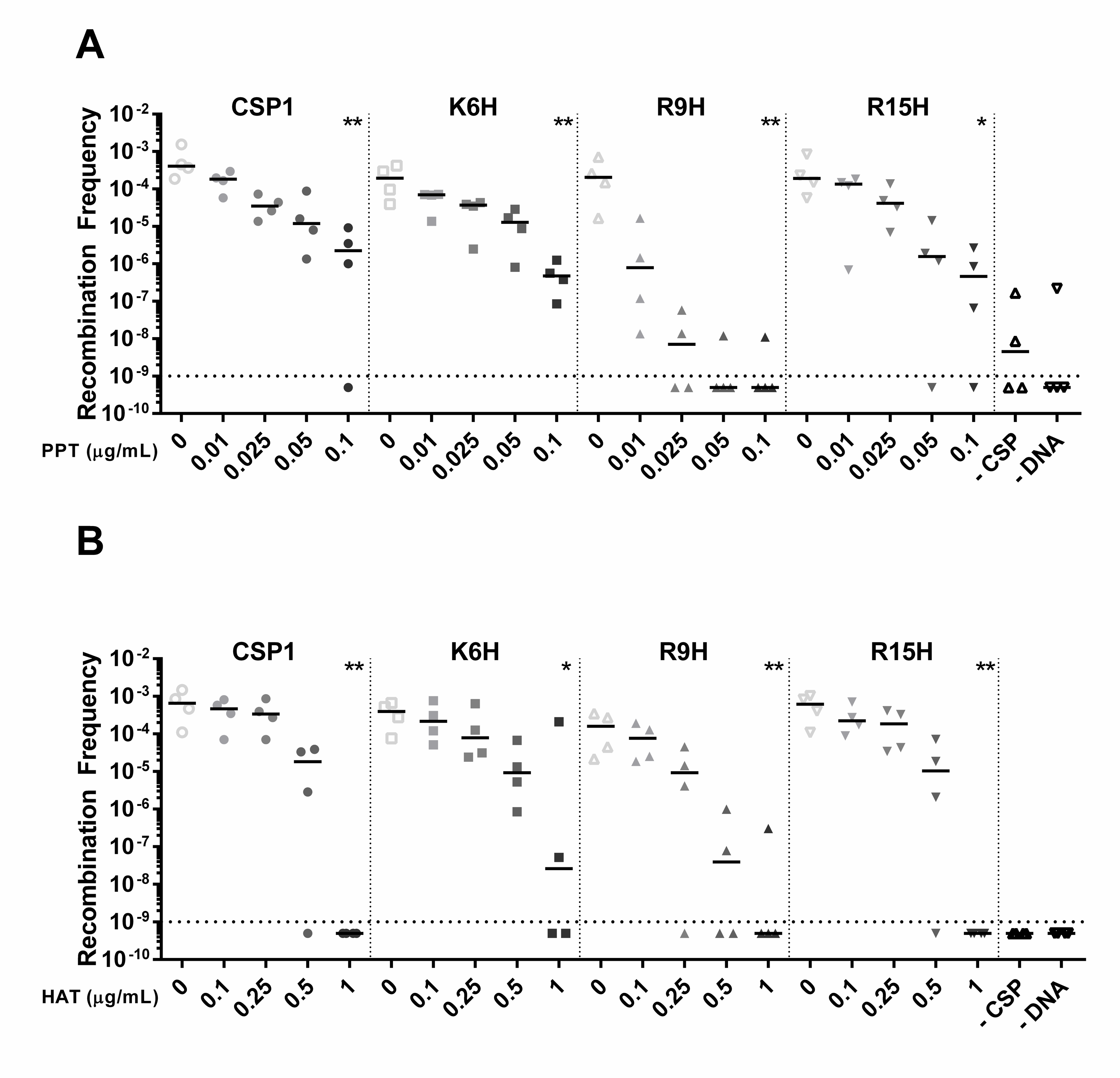

Supplement: S2 Fig — Recombination frequency upon incubation of modified CSP1 with increasing concentrations of serine proteases. Transformation of D39x comC- with modified CSP1 incubated with (A) PPT or (B) HAT. Negative controls included no addition of CSP1 and no addition of gDNA. Lines represent median value. Dotted line represents lowest point of detection. Recombination frequencies of increasing concentrations of protease within each modified CSP were compared using Kruskal-Wallis one-way ANOVA; *p = 0.05–0.01, **p = 0.01–0.001. Changes in the concentrations of protease between modified CSP was compared using two-way ANOVA; all groups non-significant. (TIF) [file ppat.1011421.s002.tif]

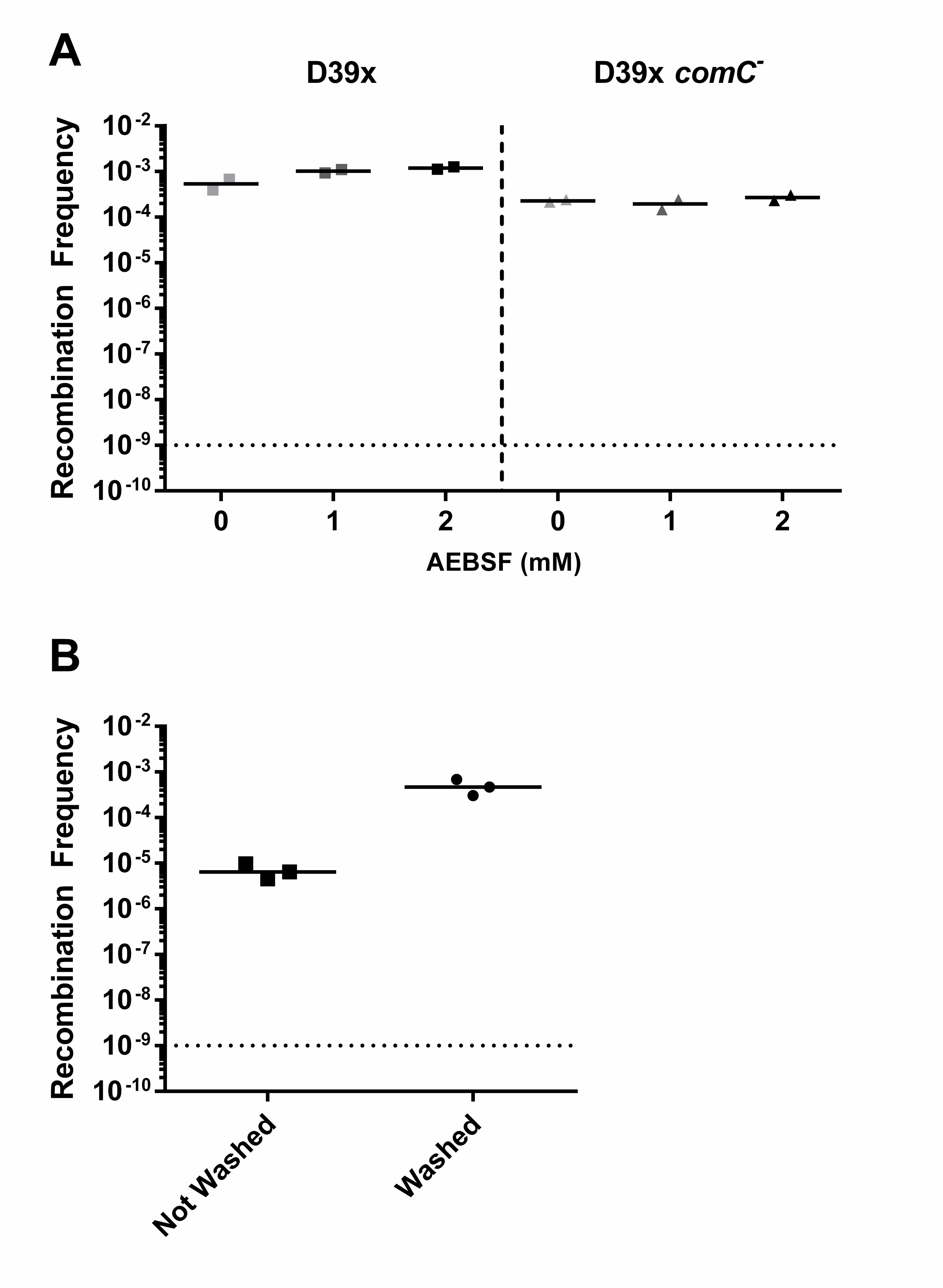

Supplement: S3 Fig — (A) Recombination frequency of D39x and D39x comC- upon incubation of CSP1 in PBS with increasing concentrations of AEBSF. Lines represent median. Dotted line represents lowest point of detection. (B) Recombination frequency of D39x comC- with and without washing cells with fresh media. Lines represent median. Dotted line represents lowest point of detection. (TIF) [file ppat.1011421.s003.tif]

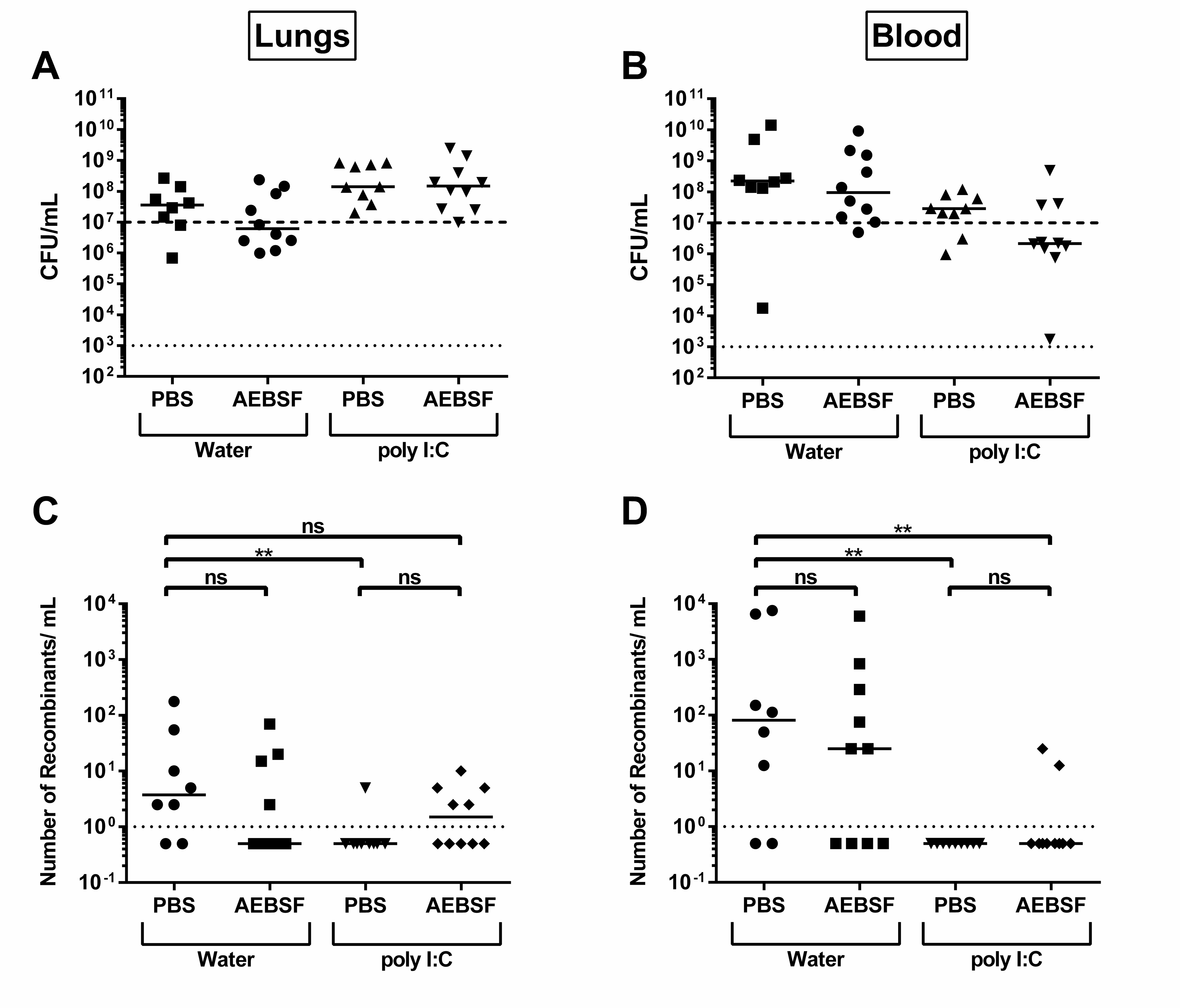

Supplement: S4 Fig — Total number of cells per mL recovered from (A) the lungs and (B) the blood of mice that received no stimulant (water) or poly (I:C), and either received no inhibitor (PBS) or inhibitor AEBSF. Lines represent median. Dashed line at 107 represents threshold for detection of recombinants. Any bacterial burden that fell below this threshold was excluded from Fig 8. Dotted line represents lowest point of detection. Total number of recombinants per mL recovered from (C) the lungs and (D) the blood. Lines represent median. Number of recombinants per mL of all groups were compared pairwise for each tissue using nonparametric Mann-Whitney t test; **p = 0.01–0.001. (TIF) [file ppat.1011421.s004.tif]
